# Redox-neutral and metal-free synthesis of 3-(arylmethyl)chroman-4-ones *via* visible-light-driven alkene acylarylation

**DOI:** 10.3389/fchem.2022.1059792

**Published:** 2022-10-31

**Authors:** Yan Ding, Shengjiao Yu, Man Ren, Ji Lu, Qiang Fu, Zhijie Zhang, Qin Wang, Jun Bai, Na Hao, Lin Yang, Siping Wei, Dong Yi, Jun Wei

**Affiliations:** ^1^ Central Nervous System Drug Key Laboratory of Sichuan Province, Department of Medicinal Chemistry, School of Pharmacy, Southwest Medical University, Luzhou, China; ^2^ Department of Chemistry, School of Basic Medical Sciences, Southwest Medical University, Luzhou, China; ^3^ School of Public Health, Southwest Medical University, Luzhou, China

**Keywords:** chroman-4-one, 3-(arylmethyl)chroman-4-ones, phosphoranyl radical, acyl radical, radical-radical coupling

## Abstract

A metal- and aldehyde-free visible-light-driven photoredox-neutral alkene acylarylation with readily available cyanoarenes is described. A variety of 3-(arylmethyl)chroman-4-ones (i.e., homoisoflavonoids) and analogs are efficiently synthesized with good functional group tolerance. This mild protocol relies on a phosphoranyl radical-mediated acyl radical-initiated cyclization and selective radical-radical coupling sequence, and is also further highlighted by subsequent derivatization to chromone and 2*H*-chromene as well as its application in the three-component alkene acylarylation.

## Introduction

**FIGURE 1:**
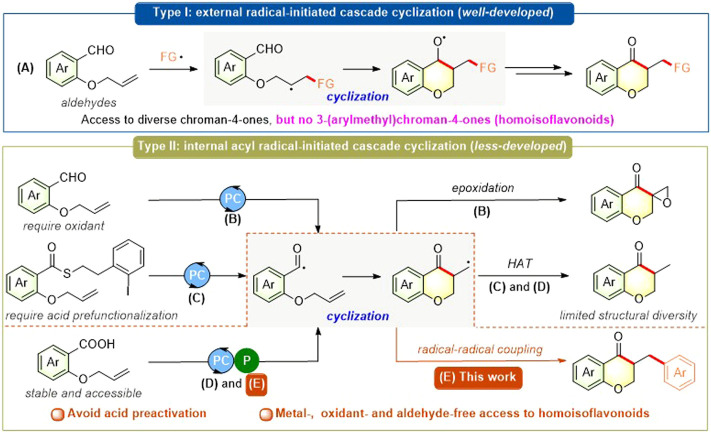
Photocatalytic radical-initiated cascade cyclization toward functionalized chroman-4-ones.

Chroman-4-one scaffolds, a class of important oxygen-containing structural motifs, are ubiquitous in a plethora of natural products, drug candidates, and biologically active molecules (Albrecht et al., 2005; Nibbs and Scheidt, 2011; Friden-Saxin et al., 2012; Lee et al., 2014; Seifert et al., 2014; Emami and Ghanbarimasir, 2015; Kumar et al., 2017; Mayuri et al., 2017). In the past years, the radical-initiated cascade cyclization strategy has attracted great attention for the construction of chroman-4-one scaffold and other (hetero)cyclic frameworks (Zhao et al., 2016; Yang et al., 2017; Hu et al., 2018a; Hu et al., 2018b; Liu et al., 2019; Sheng et al., 2019; Xiao et al., 2019; Zhou et al., 2019a; Das et al., 2020; Han et al., 2020; Huang et al., 2020; Liu et al., 2020; Mei et al., 2020; Xiong et al., 2020; Diana et al., 2021). Particularly, the photocatalytic radical-initiated cascade cyclization, including two mechanistically distinctive pathways, has emerged as an elegant, green, and powerful strategy for the synthesis of such scaffold and its derivatives. The first photocatalytic approach to diversely functionalized chroman-4-ones *via* various external radical-initiated cascade cyclization of *o*-(allyloxy) arylaldehydes is well-developed by the groups of Zhu (Lu et al., 2017; Zhou et al., 2019b), Yu (Zhu et al., 2021), Xuan (He et al., 2019), and others (Zhou et al., 2019a; Huang et al., 2020; Mei et al., 2020; Liu et al., 2022) (Figure 1A). In contrast, there are only a few examples of photocatalytic internal acyl radical-initiated cascade cyclization (Jung et al., 2017; Norman et al., 2018; Stache et al., 2018; Zhou et al., 2021) (Figures 1B–D), which limits their application for the rapid assembly of structurally diverse chroman-4-ones. Recently, Hong group (Jung et al., 2017) and Wan group (Zhou et al., 2021) independently developed a visible-light-driven radical cyclization/epoxidation of *o*-(allyloxy)arylaldehydes toward spiroepoxy chroman4-one scaffolds using Ru (bpy)_3_Cl_2_ or organoselenium as photocatalyst and *tert*-butyl hydroperoxide (TBHP) as oxidant (Figure 1B). In 2018, McErlean group (Norman et al., 2018) disclosed a photoredox-catalyzed indirect acyl radical generation from relatively stable Crich-type thioesters generated in a single step with carboxylic acid starting materials, followed by intramolecular alkene addition/cyclization to give various cyclic ketones including chroman-4-one scaffold (Figure 1C). However, these existing strategies are solely based on the elaboration of uneasily available *o*-(allyloxy)arylaldehydes (almost all) or carboxylic acid thioesters (only one) and also suffer from one or more drawbacks such as excess amounts of oxidants, limited structural diversity, and lack of functionality tolerance. Therefore, the development of alternative and efficient approaches to access diversely functionalized chroman-4-one and related cyclic ketone analogs *via* photocatalytic internal acyl radical-initiated cascade cyclization using accessible starting materials should be highly desirable.

Carboxylic acids as starting materials are not only abundant, generally stable, and readily accessible in great structural diversity, and have also drawn much attention for their application as versatile radical precursors such as alkyl, aryl, carboxylic, and particularly acyl radicals (Mandal et al., 2018; Wang et al., 2019; Hu et al., 2020b; Chan et al., 2022; Kitcatt et al., 2022; Yan et al., 2022). Recently, an elegant strategy that combines photoredox catalysis and phosphoranyl radical-mediated deoxygenation makes it possible to form acyl radicals from carboxylic acids, providing streamlined access to structurally diverse ketones (Zhang et al., 2017; Stache et al., 2018). However, to the best of our knowledge, there are not only a few reports on the application of this powerful strategy to intermolecular and intramolecular alkene acylations including *ipso*-acylation (Li et al., 2022b), defluorinative acylation (Guo et al., 2020), and hydro-acylation (one example of hydro-acylation: one compound chroman-4-one using expensive iridium-based photocatalyst, Figure 1D) (Stache et al., 2018; Zhang et al., 2018; Martinez Alvarado et al., 2019; Merkens et al., 2021), but also no report on alkene difuntionalizations (especially carbon-acylation) with this strategy to date. Inspired by these work and seminal pioneering reports on the photoredox-catalyzed radical-type *ipso*-functionalizations of electron-deficient cyanoarene derivatives (Betori and Scheidt, 2019; Vorob’ev, 2019; Zhong et al., 2020; Zhou et al., 2020; Shen et al., 2021; Tong et al., 2021; Georgiou et al., 2022), we envisaged whether the radical relay strategy of the phosphoranyl radical-mediated acyl radical-initiated cascade cyclization from alkene-tethered carboxylic acids and subsequent radical-radical coupling process could enable the rapid construction of 3-(arylmethyl)chroman-4-ones, which are one of the core frameworks in a variety of homoisoflavonoids with various biological activities (Eggler et al., 1991; Desideri et al., 1997; Eggler et al., 1997; Tait et al., 2006; Basavarajappa et al., 2015). Herein, we report an efficient and practical approach for the metal-, oxidant-, and aldehyde-free synthesis of 3-(arylmethyl)chroman-4-ones and other cyclic ketone analogs via visible light-driven photoredox-neutral alkene acylarylation (being a class of alkene carbon-acylation, Figure 1E).

## Results and discussion

To corroborate this hypothesis, we initially selected a model reaction of alkenoic acid **1a** and 4-cyanopyridine **2a** to explore the reaction conditions under 30 W blue LED irradiation at room temperature (Table 1). To our delight, the desired 3-(pyridylmethyl)chroman-4-one **3aa** could be obtained in 75% yield by using 3DPAFIPN as a metal-free photocatalyst (entry 1). In light of the fact that the excited state *3DPAFIPN [*E*_1/2_ (PC^*^/PC^•−^) = +1.09 V *vs.* SCE] is a strong oxidant (Speckmeier et al., 2018), single electron transfer (SET) could occur from P (*p*-tol)_3_ (*E*_1/2_^ox^ = +1.03 V *vs.* SCE, Supplementary Figure S3) to *3DPAFIPN. Additionally, the presence of alkenoic acid **1a** shifted the reductive potential of **2a** from −1.81 V vs. SCE to −1.33 V vs. SCE (Supplementary Figure S4), thus enabling SET between the reduced 3DPAFIPN^•−^ [*E*_1/2_ (PC/PC_red_) = −1.59 V vs*.* SCE] and **2a** to complete the photocatalytic cycle without any aid of external reductant and oxidant. Next, decreasing the loading of the photosensitizer from 2 mol% to 1 mol% or 0.5 mol% obtained a slightly decreasing yield (entry 1). Other organic photosensitizers such as 3DPA2FBN with suitable oxidative-reductive potential (Speckmeier et al., 2018) could also afford the desired chroman-4-one in good yields, while using 4CzIPN (Speckmeier et al., 2018) led to no desired product (entries 2–3). Furthermore, other electron-rich trivalent phosphorus compounds could also be used as the phosphorus source in this deoxygenative transformation (entries 4–8), whereas using relatively electron-deficient ones instead of P (*p*-tol)_3_ led to poor efficiency (entries 9–11). Then, the screening of solvents demonstrated that these photocatalytic reactions performed in CH_2_Cl_2_ or DCE also resulted in synthetically useful yields, while other solvents such as DMF, DMSO, and THF gave no desired product (entries 12–14). Further control experiments performed in the absence of light, photocatalyst, or phosphine failed to give the desired chroman-4-one, thus emphasizing their crucial role in this photocatalytic acylarylation (entry 15).

**TABLE 1:** Optimization of the reaction conditions^a^ ^,^ ^b^.

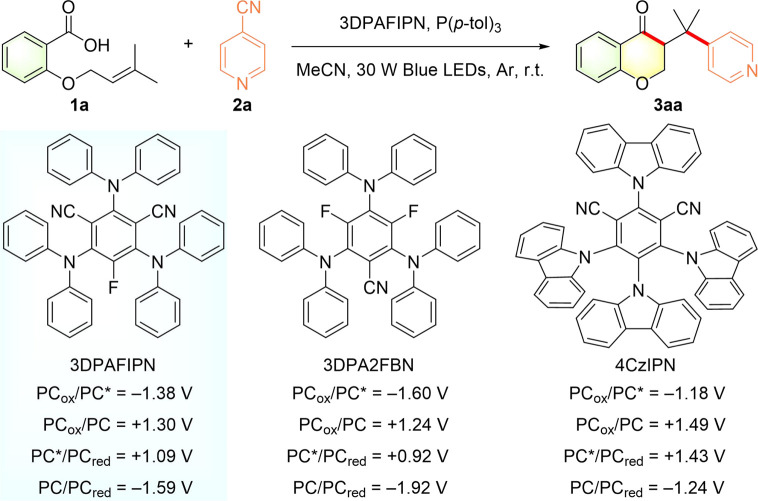		
Entry	Variation from the Standard Conditions	Yield
1	none	75%, 67%^c^, 53%^d^
2	3DPA2FBN instead of 3DPAFIPN	66%
3	4CzIPN instead of 3DPAFIPN	n.d
4	PPh_3_ instead of P(*p*-tol)_3_	65%
5	PMePh_2_ instead of P(*p*-tol)_3_	53%
6	P(*p*-tol)Ph_2_ instead of P(*p*-tol)_3_	63%
7	P(*p*-MeO-C_6_H_4_)_3_ instead of P(*p*-tol)_3_	63%
8	P(*o*-MeO-C_6_H_4_)_3_ instead of P(*p*-tol)_3_	62%
9	P(C_6_F_5_)_3_ instead of P(*p*-tol)_3_	Trace
10	P(OEt)Ph_2_ instead of P(*p*-tol)_3_	17%
11	P(OEt)_3_ instead of P(*p*-tol)_3_	Trace
12	CH_2_Cl_2_ instead of MeCN	42%
13	DCE instead of MeCN	47%
14	DMF, DMSO, or THF instead of MeCN	n.d
15	no light or photocatalyst or P(*p*-tol)_3_	n.d

a Reaction conditions: **1a** (0.1 mmol), **2a** (0.15 mmol), 3DPAFIPN (2 mol%), phosphine (0.2 mmol), solvent (2 ml), 30 W blue LEDs, argon atmosphere, r.t., 24 h; n.d. = not detected.

^b^ Yields were determined by ^1^H NMR, using 1,3,5-trimethoxybenzene as an internal standard.

^c^ 1 mol% 3DPAFIPN.

^d^ 0.5 mol% 3DPAFIPN.

**FIGURE 2:**
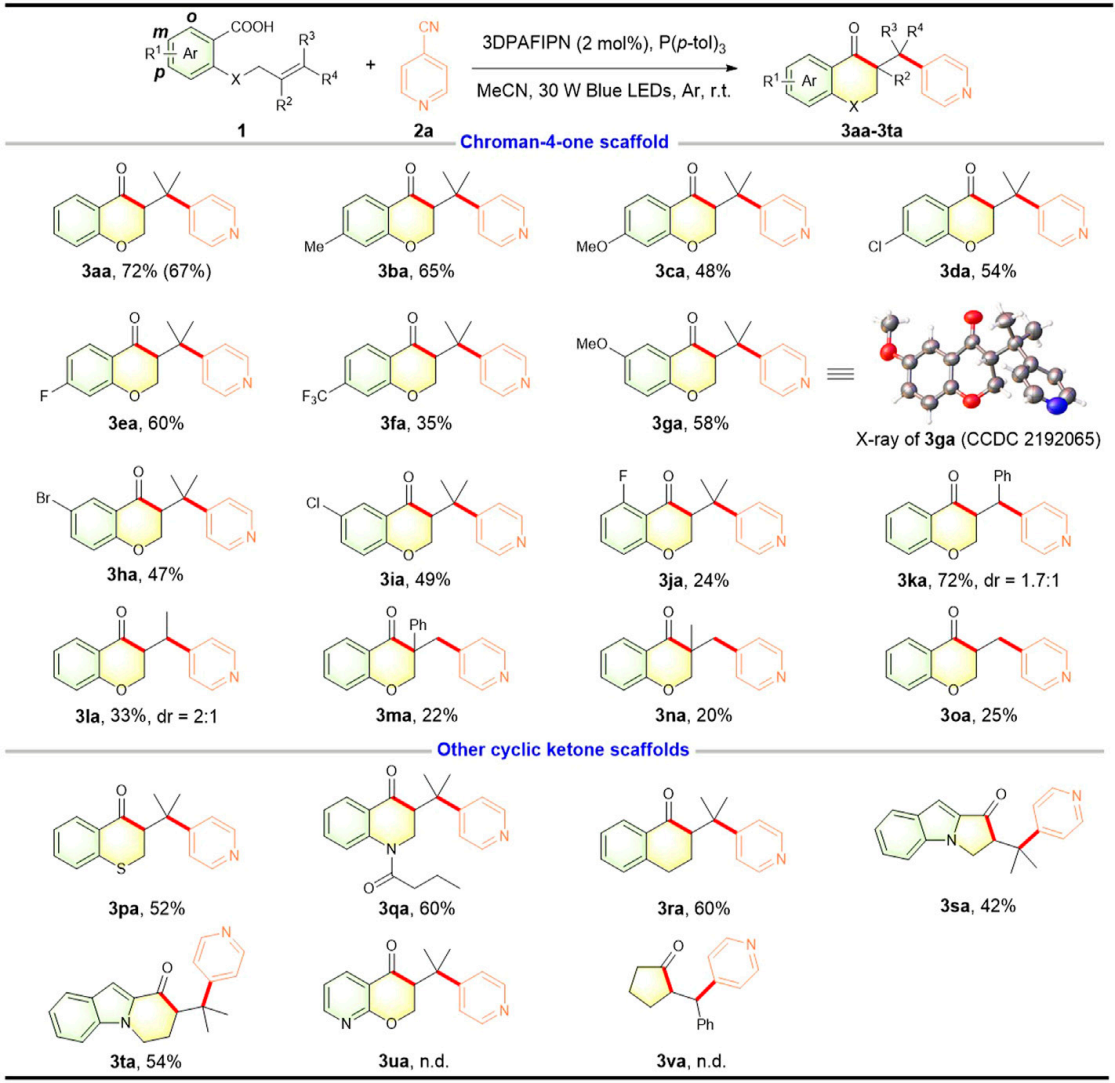
Scope of alkenoic acids. Reaction conditions: **1** (0.3 mmol), **2a** (0.45 mmol), 3DPAFIPN (2 mol%), P (*p*-tol)_3_ (0.6 mmol), MeCN (6 ml), 30 W blue LEDs, argon atmosphere, r.t., 24 h. The isolated yield is based on **1**. Isolated yield in parentheses is obtained on a 1.0 mmol scale.

With the optimized reaction conditions in hand, we investigated the scope and limitations of this reaction using a variety of alkenoic acids (Figure 2). It was worth mentioning that this photocatalytic reaction could be run on a 1.0 mmol scale to provide the target product **3aa** in 67% yield. Firstly, we examined the effect of the aromatic moiety of the substrate alkenoic acid. It was found that the electron-donating group (Me, OMe) and electron-withdrawing groups (F, Cl, Br, CF_3_) at the *para*- and *mata*-position with respect to the carboxylic acid were well compatible with this transformation and the corresponding chroman-4-ones were obtained with satisfactory yields (**3ba**–**3ia**). The structure of **3ga** was confirmed by X-ray diffraction analysis (CCDC 2192065). Moreover, the *ortho*-F substituted alkenoic acid was also employed in this transformation, providing the desired chroman-4-one **3ja** albeit in a relatively low yield. Then, we investigated the scope of the alkene moiety of the substrate alkenoic acid. 1,2-Disubstituted nonterminal alkenoic acid with a phenyl group at the terminal carbon participated well in such acylarylation to give the expected product **3ka**, while one with an alkyl group was transformed into the compound **3la** with a low yield. And 1,1-disubstituted or mono-substituted terminal alkenoic acid could also be subjected to this transformation, affording the corresponding chroman-4-ones (**3ma**–**3oa**) albeit with diminished yields. Interestingly, replacing the oxygen atom at the *ortho*-position with respect to the carboxylic acid by an atom of sulphur, nitrogen, or carbon favored the photocatalytic acylarylation, leading to the corresponding chroman-4-one analogs such as thiochroman-4-one **3pa**, dihydroquinolin-4(1*H*)-one **3qa**, and dihydronaphthalen-1(2*H*)-one **3ra**. Additionally, *N*-(homo)allylindole-2-carboxylic acids were proved to be suitable heteroaromatic substrates for this photocatalytic process and gave the architecturally intriguing and valuable tricyclic ketone framework including 1*H*-pyrrolo [1,2-*a*]indol-1-one (**3sa**) and pyrido [1,2-*a*]indol-9(6*H*)-one (**3ta**) in comparable yields. These experimental outcomes fully highlighted the synthetic potential to construct structurally complex ketone-containing (hetero)cycles. However, pyridyl-substituted alkenoic acid **1u** and acyclic aliphatic alkenoic acid **1v** could be not suitable for this alkene acylarylation.

**FIGURE 3:**
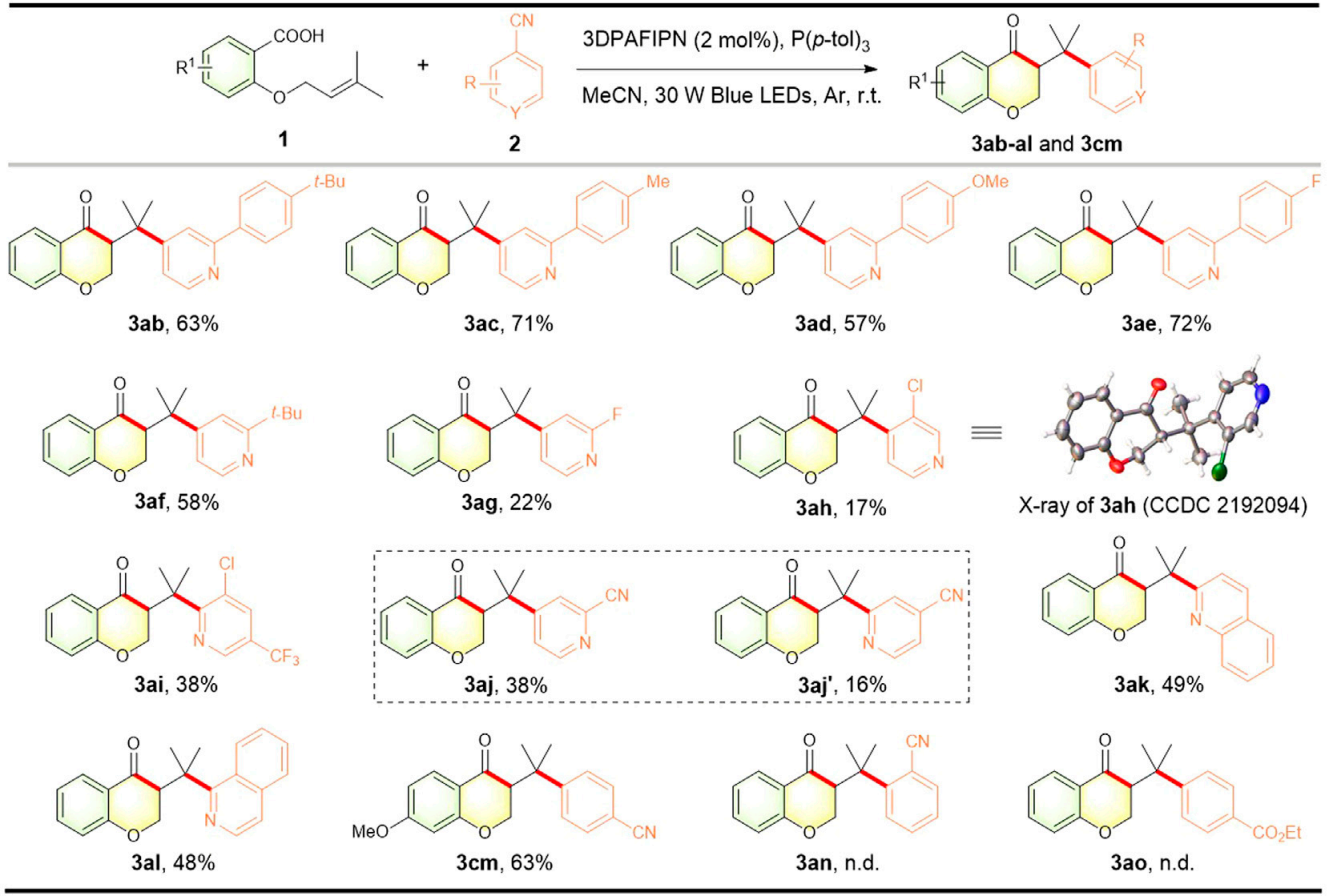
Scope of cyano (hetero)arenes. Reaction conditions: **1** (0.3 mmol), **2** (0.45 mmol), 3DPAFIPN (2 mol%), P (*p*-tol)_3_ (0.6 mmol), MeCN (6 ml), 30 W blue LEDs, argon atmosphere, r.t., 24 h. The isolated yield is based on **1**.

To further explore the synthetic potential of our methodology, we then investigated differently substituted cyanoarene partners in this photocatalytic acylarylation (Figure 3). Firstly, the substituted phenyl and alkyl group at the 2-position of cyanopyridine were well tolerated, providing the corresponding 3-(pyridylmethyl)chroman-4-ones (**3ab**–**3af**) in moderate to good yields. Cyanopyridines bearing halogen substituents at 2- or 3-position afforded the desired products albeit in decreased yields (**3ag**–**3ai**), offering opportunities for further derivatization. The structure of **3ah** was confirmed by X-ray diffraction analysis (CCDC 2192094). Notably, using 2, 4-dicyanopyridine as a coupling partner underwent selective coupling at the most electron-poor 4-position to produce the corresponding chroman-4-one **3aj** in a synthetically useful yield along with C2-coupled chroman-4-one **3aj′**. Additionally, non-pyridine cyanoarenes including quinoline and isoquinoline scaffolds were also successful with the standard conditions, leading to the formation of the corresponding chroman-4-ones **3ak** and **3al** with 49% and 48% yields, respectively. To our delight, other electron-withdrawing cyanoarene 1, 4-dicyanobenzene was also compatible with our protocol to give a satisfactory yield of 3-benzylchroman-4-one **3cm** (belonging to classical homoisoflavonoid skeleton), while 1,2-dicyanobenzene **2n** and ethyl 4-cyanobenzoate **2o** were not suitable.

**FIGURE 4:**
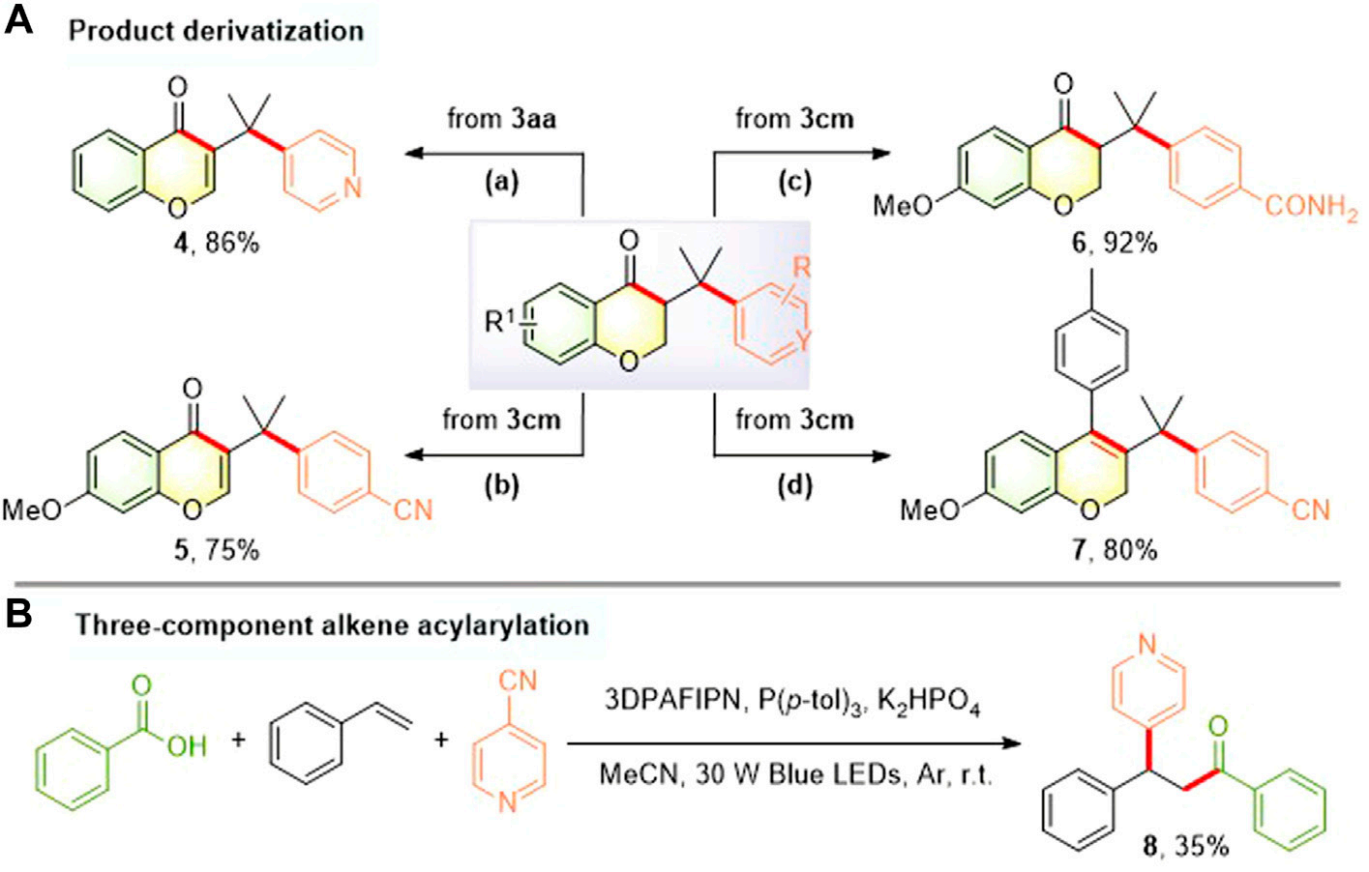
Product derivatization **(A)** and three-component alkene acylarylation **(B)**. Reaction conditions: (a) I_2_, DMSO, reflux 2 h (b) I_2_, DMSO, reflux 2 h (c) 30% H_2_O_2_, K_2_CO_3_, DMSO, 0°C to r.t., 24 h (d) 2,6-di-*tert*-butylpyridine, Tf_2_O, CH_2_Cl_2_, 0°C to r.t., 5 h; Pd(PPh_3_)_4_, *p*-tolylboronic acid, DIPEA, NMP, 170°C, 10 min.

To investigate the practical utility of this photocatalytic acylarylation process, several illustrative examples of simple derivatization of 3-(arylmethyl)chroman-4-ones were provided (Figure 4). For example, I_2_-mediated dehydrogenation of the resulting 3-(arylmethyl)chroman-4-ones (**3aa** and **3cm**) proceeded well to provide the extensively studied and medicinally important chromones [**4** (Zheng et al., 2015; Gobbi et al., 2016) and **5** (Kirkiacharian et al., 1989; Cavalli et al., 2005; Kirkiacharian and Gomis, 2005; Rao et al., 2008; Kupcewicz et al., 2013; Noshita et al., 2021)] with 86% and 75% yields, respectively. 3-Benzylchroman-4-one **3cm** was treated with H_2_O_2_ in the presence of K_2_CO_3_ to obtain the corresponding amide **6** in 92% yield. Moreover, elaborated alkenyl triflate derived from **3cm** could undergo Pd-mediated Suzuki coupling to afford biologically intriguing 3-benzyl 2*H*-chromene (Srikanth et al., 1997; Conti and Desideri, 2009) **7** in 80% yield. To our delight, the first example for the more challenging three-component alkene acylarylation using simple and easily accessible feedstocks could be realized to afford *β*-pyridylated ketone **8** albeit in a relatively low yield (Figure 4B), which is complementary to the previously reported two-component synthesis of pyridyl-containing ketones (Zhu et al., 2020; Yang et al., 2021; Li et al., 2022a).

**FIGURE 5:**
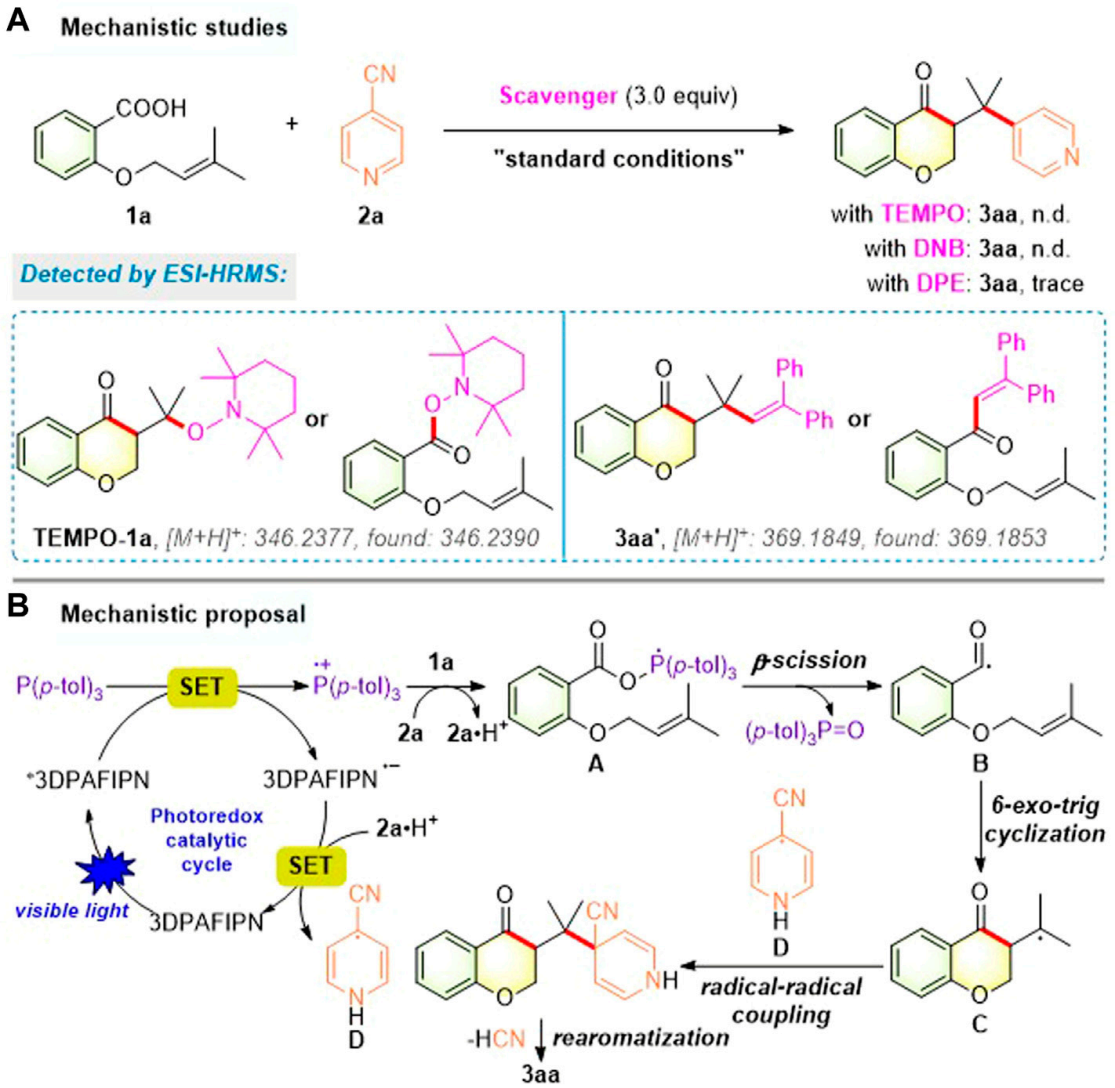
Mechanistic studies **(A)** and mechanistic proposal **(B)**.

To elucidate the mechanism of this photocatalytic acylarylation, several control experiments using substrates **1a** and **2a** were carried out as shown in Figure 5A. When three equivalents of the radical scavenger TEMPO or the electron-transfer scavenger *p*-dinitrobenzene (DNB) were added under standard conditions, no product **3aa** was observed and the corresponding TEMPO-adduct (**TEMPO-1a**) was detected by ESI-HRMS analysis. Additionally, when the model reaction was performed with an external radical-trapping reagent 1,1-diphenylethylene (DPE), the formation of the desired chroman-4-one **3aa** was significantly inhibited and the corresponding radical-trapping product **3aa′** was also detected by ESI-HRMS analysis. Taken together, these results indicate that a radical/SET-based pathway might be involved in our photocatalytic acylarylation.

Based on the above experimental results and previous reports (Jiang et al., 2019; Hu et al., 2020a; Clarke et al., 2020; Nicastri et al., 2020; Pan et al., 2020; Rossi-Ashton et al., 2020; Shao et al., 2020; Zhou et al., 2020; Tong et al., 2021), a plausible mechanistic pathway for this photocatalytic acylarylation is proposed as described in Figure 5B. Under the blue LED irradiation, the photocatalyst 3DPAFIPN was initially raised to the excited state *3DPAFIPN, which was reductively quenched by P (*p*-tol)_3_ to form the strongly reducing 3DPAFIPN^•−^ and phosphine radical cation. Subsequently, the phosphine radical cation recombined with the carboxylate anion of **1a** to produce the phosphoranyl radical intermediate **A**, which underwent a facile *β*-scission to form acyl radical **B** and tri-*p*-tolylphosphine oxide. Then, the resulting radical **B** proceeded *via* intramolecular 6-exo-trig cyclization with the alkene moiety to provide alkyl radical **C**. Meanwhile, SET between the reduced 3DPAFIPN^•−^ and **2a⋅H** ^+^ gave a persistent arene radical **D** and regenerated 3DPAFIPN. Finally, the alkyl radical **C** underwent intermolecular radical-radical coupling with radical **D** and sequential rearomatization *via* the elimination of both cyano anion and proton to achieve the corresponding chroman-4-one **3aa**.

## Conclusion

In summary, we have developed a novel visible-light-driven photoredox-neutral alkene acylarylation with cyanoarenes, enabling metal-, oxidant-, and aldehyde-free access to structurally diverse 3-(arylmethyl)chroman-4-ones (i.e., homoisoflavonoids) as well as other cyclic ketone analogs such as thiochroman-4-one, dihydroquinolin-4(1*H*)-one, dihydronaphthalen-1(2*H*)-one, pyrrolo [1,2-*a*]indol-1-one, and pyrido [1,2-*a*]indol-9(6*H*)-one. Furthermore, the resulting chroman-4-ones can be scale-up synthesized and also readily parlayed into skeletally diverse and valuable compounds such as chromone and 2*H*-chromene. In addition, the developed powerful protocol involves phosphoranyl radical-mediated acyl radical-initiated cascade cyclization followed by radical-radical coupling with the persistent aryl radical, enabling the concomitant introduction of ketone and aromatic fragments to organic molecules.

## Data Availability

The datasets presented in this study can be found in online repositories. The names of the repository/repositories and accession number(s) can be found below: https://www.ccdc.cam.ac.uk/structures/- CCDC 2192065.

## References

[B1] Albrecht U. Lalk M. Langer P. (2005). Synthesis and structure-activity relationships of 2-vinylchroman-4-ones as potent antibiotic agents. Bioorg. Med. Chem. 13, 1531–1536. 10.1016/j.bmc.2004.12.031 15698769

[B2] Basavarajappa H. D. Lee B. Lee H. Sulaiman R. S. An H. Magana C. (2015). Synthesis and biological evaluation of novel homoisoflavonoids for retinal neovascularization. J. Med. Chem. 58, 5015–5027. 10.1021/acs.jmedchem.5b00449 26035340 PMC4944207

[B3] Betori R. C. Scheidt K. A. (2019). Reductive arylation of arylidene malonates using photoredox catalysis. ACS Catal. 9, 10350–10357. 10.1021/acscatal.9b03608 36247345 PMC9564127

[B4] Cavalli A. Bisi A. Bertucci C. Rosini C. Paluszcak A. Gobbi S. (2005). Enantioselective nonsteroidal aromatase inhibitors identified through a multidisciplinary medicinal chemistry approach. J. Med. Chem. 48, 7282–7289. 10.1021/jm058042r 16279787

[B5] Chan A. Y. Perry I. B. Bissonnette N. B. Buksh B. F. Edwards G. A. Frye L. I. (2022). Metallaphotoredox: The merger of photoredox and transition metal catalysis. Chem. Rev. 122, 1485–1542. 10.1021/acs.chemrev.1c00383 34793128 PMC12232520

[B6] Clarke A. K. Parkin A. Taylor R. J. K. Unsworth W. P. Rossi-Ashton J. A. (2020). Photocatalytic deoxygenation of sulfoxides using visible light: Mechanistic investigations and synthetic applications. ACS Catal. 10, 5814–5820. 10.1021/acscatal.0c00690 32582464 PMC7304878

[B7] Conti C. Desideri N. (2009). Synthesis and antirhinovirus activity of new 3-benzyl chromene and chroman derivatives. Bioorg. Med. Chem. 17, 3720–3727. 10.1016/j.bmc.2009.03.051 19398344

[B8] Das S. Parida S. K. Mandal T. Sing L. De Sarkar S. Murarka S. (2020). Organophotoredox-catalyzed cascade radical annulation of 2-(allyloxy)arylaldehydes with N-(acyloxy)phthalimides: Towards alkylated chroman-4-one derivatives. Chem. Asian J. 15, 568–572. 10.1002/asia.201901735 32017417

[B9] Desideri N. Olivieri S. Stein M. Sgro R. Orsi N. Conti C. (1997). Synthesis and anti-picornavirus activity of homo-isoflavonoids. Antivir. Chem. Chemother. 8, 545–555. 10.1177/095632029700800609

[B10] Diana E. J. Kanchana U. S. Mathew T. V. (2021). Current developments in the synthesis of 4-chromanone-derived compounds. Org. Biomol. Chem. 19, 7995–8008. 10.1039/d1ob01352a 34494068

[B11] Eggler J. F. Marfat A. Melvin L. S. Jr. (1991). Substituted tetralins, chromans and related compounds in the treatment of asthma, arthritis and related diseases. Washington, DC, U.S: Patent and Trademark Office. U.S. Patent NO 5,059,609.

[B12] Eggler J. F. Marfat A. Melvin L. S. Jr. (1997). Tetralin and chroman derivatives useful in the treatment of asthma, arthritis and related diseases. Washington, DC, U.S: Patent and Trademark Office. U.S. Patent NO 5,698,550.

[B13] Emami S. Ghanbarimasir Z. (2015). Recent advances of chroman-4-one derivatives: Synthetic approaches and bioactivities. Eur. J. Med. Chem. 93, 539–563. 10.1016/j.ejmech.2015.02.048 25743215

[B14] Friden-Saxin M. Seifert T. Landergren M. R. Suuronen T. Lahtela-Kakkonen M. Jarho E. M. (2012). Synthesis and evaluation of substituted chroman-4-one and chromone derivatives as sirtuin 2-selective inhibitors. J. Med. Chem. 55, 7104–7113. 10.1021/jm3005288 22746324 PMC3426190

[B15] Georgiou E. Spinnato D. Chen K. Melchiorre P. Muniz K. (2022). Switchable photocatalysis for the chemodivergent benzylation of 4-cyanopyridines. Chem. Sci. 13, 8060–8064. 10.1039/d2sc02698h 35919417 PMC9278488

[B16] Gobbi S. Hu Q. Zimmer C. Engel M. Belluti F. Rampa A. (2016). Exploiting the chromone scaffold for the development of inhibitors of corticosteroid biosynthesis. J. Med. Chem. 59, 2468–2477. 10.1021/acs.jmedchem.5b01609 26938274

[B17] Guo Y. Q. Wang R. Song H. Liu Y. Wang Q. (2020). Visible-light-Induced deoxygenation/defluorination protocol for synthesis of γ, γ-difluoroallylic ketones. Org. Lett. 22, 709–713. 10.1021/acs.orglett.9b04504 31909628

[B18] Han Q.-Q. Li G.-H. Sun Y.-Y. Chen D.-M. Wang Z.-L. Yu X.-Y. (2020). Silver-catalyzed cascade radical cyclization of sodium sulfinates and o-(allyloxy)arylaldehydes towards functionalized chroman-4-ones. Tetrahedron Lett. 61, 151704–151708. 10.1016/j.tetlet.2020.151704

[B19] He X. K. Cai B. G. Yang Q. Q. Wang L. Xuan J. (2019). Visible-light-promoted cascade radical cyclization: Synthesis of 1, 4-diketones containing chroman-4-one skeletons. Chem. Asian J. 14, 3269–3273. 10.1002/asia.201901078 31464012

[B20] Hu H. Chen X. Sun K. Wang J. Liu Y. Liu H. (2018b). Silver-catalyzed decarboxylative cascade radical cyclization of *tert*-carboxylic acids and *o*-(allyloxy)arylaldehydes towards chroman-4-one derivatives. Org. Chem. Front. 5, 2925–2929. 10.1039/c8qo00882e

[B21] Hu H. Chen X. Sun K. Wang J. Liu Y. Liu H. (2018a). Silver-catalyzed radical cascade cyclization toward 1, 5-/1, 3-dicarbonyl heterocycles: An atom-/step-economical strategy leading to chromenopyridines and isoxazole-/pyrazole-containing chroman-4-ones. Org. Lett. 20, 6157–6160. 10.1021/acs.orglett.8b02627 30251870

[B22] Hu X.-Q. Hou Y.-X. Liu Z.-K. Gao Y. (2020a). Recent advances in phosphoranyl radical-mediated deoxygenative functionalisation. Org. Chem. Front. 7, 2319–2324. 10.1039/d0qo00643b

[B23] Hu X. Q. Liu Z. K. Hou Y. X. Gao Y. (2020b). Single electron activation of aryl carboxylic acids. iScience 23, 101266–101291. 10.1016/j.isci.2020.101266 32593954 PMC7327862

[B24] Huang H. L. Du J. Y. Li Q. L. Gao F. Ma C. L. (2020). Visible-light-promoted cascade radical cyclization: Synthesis of chroman-4-ones and dihydroquinolin-4-ones. J. Org. Chem. 85, 3963–3972. 10.1021/acs.joc.9b03253 32037828

[B25] Jiang H. Mao G. Wu H. An Q. Zuo M. Guo W. (2019). Synthesis of dibenzocycloketones by acyl radical cyclization from aromatic carboxylic acids using methylene blue as a photocatalyst. Green Chem. 21, 5368–5373. 10.1039/c9gc02380a

[B26] Jung S. Kim J. Hong S. (2017). Visible light-promoted synthesis of spiroepoxy chromanone derivatives via a tandem oxidation/radical cyclization/epoxidation process. Adv. Synth. Catal. 359, 3945–3949. 10.1002/adsc.201701072

[B27] Kirkiacharian B. S. Gomis M. (2005). New convenient synthesis of homoisoflavanones and (±)-di-o-methyldihydroeucomin. Synth. Commun. 35, 563–569. 10.1081/scc-200049789

[B28] Kirkiacharian S. Tongo H. G. Bastide J. Bastide P. Grenie M. M. (1989). Synthe`se et activite´s angioprotectrice, anti-allergique et antihistaminique de benzyl-3 chromones (homo-isoflavones). Eur. J. Med. Chem. 24, 541–546. 10.1016/0223-5234(89)90060-3

[B29] Kitcatt D. M. Nicolle S. Lee A. L. (2022). Direct decarboxylative giese reactions. Chem. Soc. Rev. 51, 1415–1453. 10.1039/d1cs01168e 35099488

[B30] Kumar D. Sharma P. Singh H. Nepali K. Gupta G. K. Jain S. K. (2017). The value of pyrans as anticancer scaffolds in medicinal chemistry. RSC Adv. 7, 36977–36999. 10.1039/c7ra05441f

[B31] Kupcewicz B. Balcerowska-Czerniak G. Malecka M. Paneth P. Krajewska U. Rozalski M. (2013). Structure-cytotoxic activity relationship of 3-arylideneflavanone and chromanone (e, z isomers) and 3-arylflavones. Bioorg. Med. Chem. Lett. 23, 4102–4106. 10.1016/j.bmcl.2013.05.044 23756061

[B32] Lee B. Basavarajappa H. D. Sulaiman R. S. Fei X. Seo S. Y. Corson T. W. (2014). The first synthesis of the antiangiogenic homoisoflavanone, cremastranone. Org. Biomol. Chem. 12, 7673–7677. 10.1039/c4ob01604a 25167470 PMC4167916

[B33] Li Y. Han C. Wang Y. Huang X. Zhao X. Qiao B. (2022a). Catalytic asymmetric reductive azaarylation of olefins via enantioselective radical coupling. J. Am. Chem. Soc. 144, 7805–7814. 10.1021/jacs.2c01458 35471031

[B34] Li Y. Shao Q. He H. Zhu C. Xue X. S. Xie J. (2022b). Highly selective synthesis of all-carbon tetrasubstituted alkenes by deoxygenative alkenylation of carboxylic acids. Nat. Commun. 13, 10–17. 10.1038/s41467-021-27507-x 35121730 PMC8816943

[B35] Liu Q. Lu W. Xie G. Wang X. (2020). Metal-free synthesis of phosphinoylchroman-4-ones via a radical phosphinoylation-cyclization cascade mediated by K_2_S_2_O_8_ . Beilstein J. Org. Chem. 16, 1974–1982. 10.3762/bjoc.16.164 32831954 PMC7431760

[B36] Liu Q. Xie G. Wang Q. Mo Z. Li C. Ding S. (2019). Synthesis of chroman-4-one and indanone derivatives via silver catalyzed radical ring opening/coupling/cyclization cascade. Tetrahedron 75, 130490–130498. 10.1016/j.tet.2019.130490

[B37] Liu Y. C. Chen P. Li X. J. Xiong B. Q. Liu Y. Tang K. W. (2022). Visible-light-induced dual acylation of alkenes for the construction of 3-substituted chroman-4-ones. J. Org. Chem. 87, 4263–4272. 10.1021/acs.joc.1c03100 35234478

[B38] Lu D. Wan Y. Kong L. Zhu G. (2017). Visible-light-induced tandem radical addition-cyclization of alkenyl aldehydes leading to indanones and related compounds. Org. Lett. 19, 2929–2932. 10.1021/acs.orglett.7b01162 28504882

[B39] Mandal S. Bera T. Dubey G. Saha J. Laha J. K. (2018). Uses of K_2_S_2_O_8_ in metal-catalyzed and metal-free oxidative transformations. ACS Catal. 8, 5085–5144. 10.1021/acscatal.8b00743

[B40] Martinez Alvarado J. I. Ertel A. B. Stegner A. Stache E. E. Doyle A. G. (2019). Direct use of carboxylic acids in the photocatalytic hydroacylation of styrenes to generate dialkyl ketones. Org. Lett. 21, 9940–9944. 10.1021/acs.orglett.9b03871 31750667 PMC6927213

[B41] Mayuri B. Kavitha P. Basavoju S. Bhargavi G. Reddy K. L. (2017). Synthesis, structural characterisation and biological evolution of chromanones. J. Mol. Struct. 1145, 1–9. 10.1016/j.molstruc.2017.05.013

[B42] Mei Y. Zhao L. Liu Q. Ruan S. Wang L. Li P. (2020). Synthesis of sulfone-functionalized chroman-4-ones and chromans through visible-light-induced cascade radical cyclization under transition-metal-free conditions. Green Chem. 22, 2270–2278. 10.1039/d0gc00009d

[B43] Merkens K. Aguilar Troyano F. J. Anwar K. Gomez-Suarez A. (2021). Synthesis of gamma-oxo-alpha-amino acids via radical acylation with carboxylic acids. J. Org. Chem. 86, 8448–8456. 10.1021/acs.joc.0c02951 34060842

[B44] Nibbs A. E. Scheidt K. A. (2011). Asymmetric methods for the synthesis of flavanones, chromanones, and azaflavanones. Eur. J. Org. Chem. 2012, 449–462. 10.1002/ejoc.201101228 PMC3412359 22876166

[B45] Nicastri M. C. Lehnherr D. Lam Y. H. DiRocco D. A. Rovis T. (2020). Synthesis of sterically hindered primary amines by concurrent tandem photoredox catalysis. J. Am. Chem. Soc. 142, 987–998. 10.1021/jacs.9b10871 31904228

[B46] Norman A. R. Yousif M. N. McErlean C. S. P. (2018). Photoredox-catalyzed indirect acyl radical generation from thioesters. Org. Chem. Front. 5, 3267–3298. 10.1039/c8qo00867a

[B47] Noshita T. Fujita K. Koga T. Ouchi H. Tai A. (2021). Synthesis and biological activity of (±)-7, 3′, 4′-trihydroxyhomoisoflavan and its analogs. Bioorg. Med. Chem. Lett. 31, 127674–127677. 10.1016/j.bmcl.2020.127674 33161123

[B48] Pan D. Nie G. Jiang S. Li T. Jin Z. (2020). Radical reactions promoted by trivalent tertiary phosphines. Org. Chem. Front. 7, 2349–2371. 10.1039/d0qo00473a

[B49] Rao V. M. Damu G. L. V. Sudhakar D. Siddaiah V. Rao C. V. (2008). New efficient synthesis and bioactivity of homoisoflavonoids. ARKIVOC 2008, 285–294. 10.3998/ark.5550190.0009.b28

[B50] Rossi-Ashton J. A. Clarke A. K. Unsworth W. P. Taylor R. J. K. (2020). Phosphoranyl radical fragmentation reactions driven by photoredox catalysis. ACS Catal. 10, 7250–7261. 10.1021/acscatal.0c01923 32905246 PMC7469205

[B51] Seifert T. Malo M. Kokkola T. Engen K. Friden-Saxin M. Wallen E. A. (2014). Chroman-4-one- and chromone-based sirtuin 2 inhibitors with antiproliferative properties in cancer cells. J. Med. Chem. 57, 9870–9888. 10.1021/jm500930h 25383691

[B52] Shao X. Zheng Y. Ramadoss V. Tian L. Wang Y. (2020). Recent advances in P(III)-assisted deoxygenative reactions under photochemical or electrochemical conditions. Org. Biomol. Chem. 18, 5994–6005. 10.1039/d0ob01083a 32692327

[B53] Shen J. Zhang Y. Yu Y. Wang M. (2021). Metal-free visible-light-induced photoredox-catalyzed intermolecular pyridylation/phosphinoylation of alkenes. Org. Chem. Front. 8, 901–907. 10.1039/d0qo01218a

[B54] Sheng J. Liu J. Chen L. Zhang L. Zheng L. Wei X. (2019). Silver-catalyzed cascade radical cyclization of 2-(allyloxy)arylaldehydes with cyclopropanols: Access to chroman-4-one derivatives. Org. Chem. Front. 6, 1471–1475. 10.1039/c9qo00292h

[B55] Speckmeier E. Fischer T. G. Zeitler K. (2018). A toolbox approach to construct broadly applicable metal-free catalysts for photoredox chemistry: Deliberate tuning of redox potentials and importance of halogens in donor-acceptor cyanoarenes. J. Am. Chem. Soc. 140, 15353–15365. 10.1021/jacs.8b08933 30277767

[B56] Srikanth N. Ng S.-C. Sim K.-Y. Kon O.-L. (1997). Synthesis of 3-(p-Halobenzyl)-4-aryl-2H-chromenes as selective ligands for antiestrogen-binding sites. J. Chem. Res. (S) 1997, 202–203. 10.1039/A700565B

[B57] Stache E. E. Ertel A. B. Tomislav R. Doyle A. G. (2018). Generation of phosphoranyl radicals via photoredox catalysis enables voltage-independent activation of strong C-O bonds. ACS Catal. 8, 11134–11139. 10.1021/acscatal.8b03592 31367474 PMC6668916

[B58] Tait S. Salvati A. L. Desideri N. Fiore L. (2006). Antiviral activity of substituted homoisoflavonoids on enteroviruses. Antivir. Res. 72, 252–255. 10.1016/j.antiviral.2006.07.003 16934879

[B59] Tong S. Li K. Ouyang X. Song R. Li J. (2021). Recent advances in the radical-mediated decyanative alkylation of cyano(hetero)arene. Green Synth. Catal. 2, 145–155. 10.1016/j.gresc.2021.04.003

[B60] Vorob’ev A. Y. (2019). Photocatalytic reaction of 4-cyanopyridine with tertiary amines. Chem. Heterocycl. Compd. (N. Y). 55, 90–92. 10.1007/s10593-019-02423-7

[B61] Wang X. Han Y. F. Ouyang X. H. Song R. J. Li J. H. (2019). The photoredox alkylarylation of styrenes with alkyl N-hydroxyphthalimide esters and arenes involving C-H functionalization. Chem. Commun. (Camb.) 55, 14637–14640. 10.1039/c9cc07494e 31746852

[B62] Xiao Y.-M. Liu Y. Mai W.-P. Mao P. Yuan J.-W. Yang L.-R. (2019). A novel and facile synthesis of chroman-4-one derivatives via cascade radical cyclization under metal-free condition. ChemistrySelect 4, 1939–1942. 10.1002/slct.201900147

[B63] Xiong L. Hu H. Wei C.-W. Yu B. (2020). Radical reactions for the synthesis of 3-substituted chroman-4-ones. Eur. J. Org. Chem. 2020, 1588–1597. 10.1002/ejoc.201901581

[B64] Yan Z. Sun B. Huang P. Zhao H. Ding H. Su W. (2022). Visible-light-promoted radical alkylation/cyclization of allylic amide with N-hydroxyphthalimide ester: Synthesis of oxazolines. Chin. Chem. Lett. 33, 1997–2000. 10.1016/j.cclet.2021.09.067

[B65] Yang J. Ma J. Yan K. Tian L. Li B. Wen J. (2021). Electrochemical ammonium cation-assisted hydropyridylation of ketone-activated alkenes: Experimental and computational mechanistic studies. Adv. Synth. Catal. 364, 845–854. 10.1002/adsc.202101361

[B66] Yang W.-C. Dai P. Luo K. Ji Y.-G. Wu L. (2017). Aldehydes as carbon radical acceptors: Silver nitrate catalyzed cascade decarboxylation and oxidative cyclization toward dihydroflavonoid derivatives. Adv. Synth. Catal. 359, 2390–2395. 10.1002/adsc.201601407

[B67] Zhang M. Ruzi R. Xi J. Li N. Wu Z. Li W. (2017). Photoredox-catalyzed hydroacylation of olefins employing carboxylic acids and hydrosilanes. Org. Lett. 19, 3430–3433. 10.1021/acs.orglett.7b01391 28612606

[B68] Zhang M. Xie J. Zhu C. (2018). A general deoxygenation approach for synthesis of ketones from aromatic carboxylic acids and alkenes. Nat. Commun. 9, 3517–3526. 10.1038/s41467-018-06019-1 30158628 PMC6115474

[B69] Zhao J. Li P. Li X. Xia C. Li F. (2016). Straightforward synthesis of functionalized chroman-4-ones through cascade radical cyclization-coupling of 2-(allyloxy)arylaldehydes. Chem. Commun. (Camb.) 52, 3661–3664. 10.1039/c5cc09730d 26853677

[B70] Zheng G. Zhang Z. Kang B. Yu R. Cao Y. Zhang S. (2015). Synthesis and vasodilatation of homoisoflavones. Chin. J. Org. Chem. 35, 1112–1122. 10.6023/cjoc201411033

[B71] Zhong L.-J. Wang H.-Y. Ouyang X.-H. Li J.-H. An D.-L. (2020). Benzylic C–H heteroarylation of N-(benzyloxy)phthalimides with cyanopyridines enabled by photoredox 1, 2-hydrogen atom transfer. Chem. Commun. 56, 8671–8674. 10.1039/d0cc03619f 32609113

[B72] Zhou C. Lei T. Wei X.-Z. Ye C. Liu Z. Chen B. (2020). Metal-free, redox-neutral, site-selective access to heteroarylamine via direct radical-radical cross-coupling powered by visible light photocatalysis. J. Am. Chem. Soc. 142, 16805–16813. 10.1021/jacs.0c07600 32897073

[B73] Zhou J. Huang M. Liang Y. Wan Y. (2021). A synergetic organoselenium catalytic system for constructing 4-chromanone derivatives via a tandem process under visible light radiation. ChemistrySelect 6, 5610–5613. 10.1002/slct.202101638

[B74] Zhou N. Wu M. Zhang M. Zhou X. (2019a). Visible-light-induced difluoroacetylation of o-(allyloxy)aryl-aldehydes: Access to difluoroacetylated chroman-4-ones. Asian J. Org. Chem. 8, 828–831. 10.1002/ajoc.201900121

[B75] Zhou Y. Xiong Z. Qiu J. Kong L. Zhu G. (2019b). Visible light photocatalytic acyldifluoroalkylation of unactivated alkenes for the direct synthesis of *gem*-difluorinated ketones. Org. Chem. Front. 6, 1022–1026. 10.1039/c9qo00136k

[B76] Zhu H. L. Zeng F. L. Chen X. L. Sun K. Li H. C. Yuan X. Y. (2021). Acyl radicals from alpha-keto acids: Metal-free visible-light-promoted acylation of heterocycles. Org. Lett. 23, 2976–2980. 10.1021/acs.orglett.1c00655 33780256

[B77] Zhu H. Yin L. Chang Z. Wang Y. Dou X. (2020). Rhodium-catalyzed asymmetric conjugate addition of organoboronic acids to carbonyl-activated alkenyl azaarenes. Adv. Synth. Catal. 362, 3142–3147. 10.1002/adsc.202000211

