# Supplementary material for: Redox-neutral and metal-free synthesis of 3-(arylmethyl)chroman-4-ones via visible-light-driven alkene acylarylation
Source: Front Chem. 2022 Oct 31;10:1059792. doi: 10.3389/fchem.2022.1059792 (PMC9660241; doi:10.3389/fchem.2022.1059792)

checkCIF/PLATON report

You have not supplied any structure factors. As a result the full set of tests cannot be run.

THIS REPORT IS FOR GUIDANCE ONLY. IF USED AS PART OF A REVIEW PROCEDURE FOR PUBLICATION, IT SHOULD NOT REPLACE THE EXPERTISE OF AN EXPERIENCED CRYSTALLOGRAPHIC REFEREE.

No syntax errors found. CIF dictionary Interpreting this report

Datablock: 1

Bond precision: C-C = 0.0084 A Wavelength=0.71073

Cell:	a=13.20(3)	b=10.61(2)	c=10.83(3)
	alpha=90	beta=94.76(5)	gamma=90
Temperature:	296 K		

	Calculated	Reported
Volume	1512(6)	1511(6)
Space group	P 21/c	P 21/c
Hall group	-P 2ybc	-P 2ybc
Moiety formula	C18 H19 N O3	?
Sum formula	C18 H19 N O3	C18 H19 N O3
Mr	297.34	297.34
Dx,g cm-3	1.306	1.307
Z	4	4
Mu (mm-1)	0.089	0.089
F000	632.0	632.0
F000'	632.30	
h,k,lmax	15,12,12	15,12,12
Nref	2661	2618
Tmin,Tmax	0.982,0.982	0.864,0.864
Tmin'	0.982	

Correction method= # Reported T Limits: Tmin=0.864 Tmax=0.864
AbsCorr = MULTI-SCAN

Data completeness= 0.984 Theta(max)= 24.998

R(reflections)= 0.1054(1115) wR2(reflections)= 0.2495(2618)

S = 1.150 Npar= 202

The following ALERTS were generated. Each ALERT has the format
test-name_ALERT_alert-type_alert-level.
Click on the hyperlinks for more details of the test.

Alert level C

```
PLAT026_ALERT_3_C Ratio Observed / Unique Reflections (too) Low ..        43% Check
PLAT082_ALERT_2_C High R1 Value ..................................       0.11 Report
PLAT148_ALERT_3_C s.u. on the      a    - Axis is (Too) Large ....      0.030 Ang.
PLAT148_ALERT_3_C s.u. on the      b    - Axis is (Too) Large ....     0.0200 Ang.
PLAT148_ALERT_3_C s.u. on the      c    - Axis is (Too) Large ....      0.030 Ang.
PLAT340_ALERT_3_C Low Bond Precision on  C-C Bonds ...............    0.00841 Ang.
```

Alert level G

```
PLAT767_ALERT_4_G INS Embedded LIST 6 Instruction Should be LIST 4     Please Check
PLAT793_ALERT_4_G Model has Chirality at C9          (Centro SPGR)          R Verify
PLAT883_ALERT_1_G No Info/Value for _atom_sites_solution_primary .     Please Do !
PLAT933_ALERT_2_G Number of HKL-OMIT Records in Embedded .res File          1 Note
PLAT941_ALERT_3_G Average HKL Measurement Multiplicity ...........        4.2 Low
PLAT965_ALERT_2_G The SHELXL WEIGHT Optimisation has not Converged     Please Check
PLAT967_ALERT_5_G Note: Two-Theta Cutoff Value in Embedded .res ..       50.0 Degree
```

0 **ALERT level A** = Most likely a serious problem - resolve or explain
0 **ALERT level B** = A potentially serious problem, consider carefully
6 **ALERT level C** = Check. Ensure it is not caused by an omission or oversight
7 **ALERT level G** = General information/check it is not something unexpected

1 ALERT type 1 CIF construction/syntax error, inconsistent or missing data
3 ALERT type 2 Indicator that the structure model may be wrong or deficient
6 ALERT type 3 Indicator that the structure quality may be low
2 ALERT type 4 Improvement, methodology, query or suggestion
1 ALERT type 5 Informative message, check

It is advisable to attempt to resolve as many as possible of the alerts in all categories. Often the minor alerts point to easily fixed oversights, errors and omissions in your CIF or refinement strategy, so attention to these fine details can be worthwhile. In order to resolve some of the more serious problems it may be necessary to carry out additional measurements or structure refinements. However, the purpose of your study may justify the reported deviations and the more serious of these should normally be commented upon in the discussion or experimental section of a paper or in the "special_details" fields of the CIF. checkCIF was carefully designed to identify outliers and unusual parameters, but every test has its limitations and alerts that are not important in a particular case may appear. Conversely, the absence of alerts does not guarantee there are no aspects of the results needing attention. It is up to the individual to critically assess their own results and, if necessary, seek expert advice.

Publication of your CIF in IUCr journals

A basic structural check has been run on your CIF. These basic checks will be run on all CIFs submitted for publication in IUCr journals (*Acta Crystallographica*, *Journal of Applied Crystallography*, *Journal of Synchrotron Radiation*); however, if you intend to submit to *Acta Crystallographica Section C* or *E* or *IUCrData*, you should make sure that full publication checks are run on the final version of your CIF prior to submission.

Publication of your CIF in other journals

Please refer to the *Notes for Authors* of the relevant journal for any special instructions relating to CIF submission.

PLATON version of 19/02/2022; check.def file version of 19/02/2022

Datablock 1 - ellipsoid plot

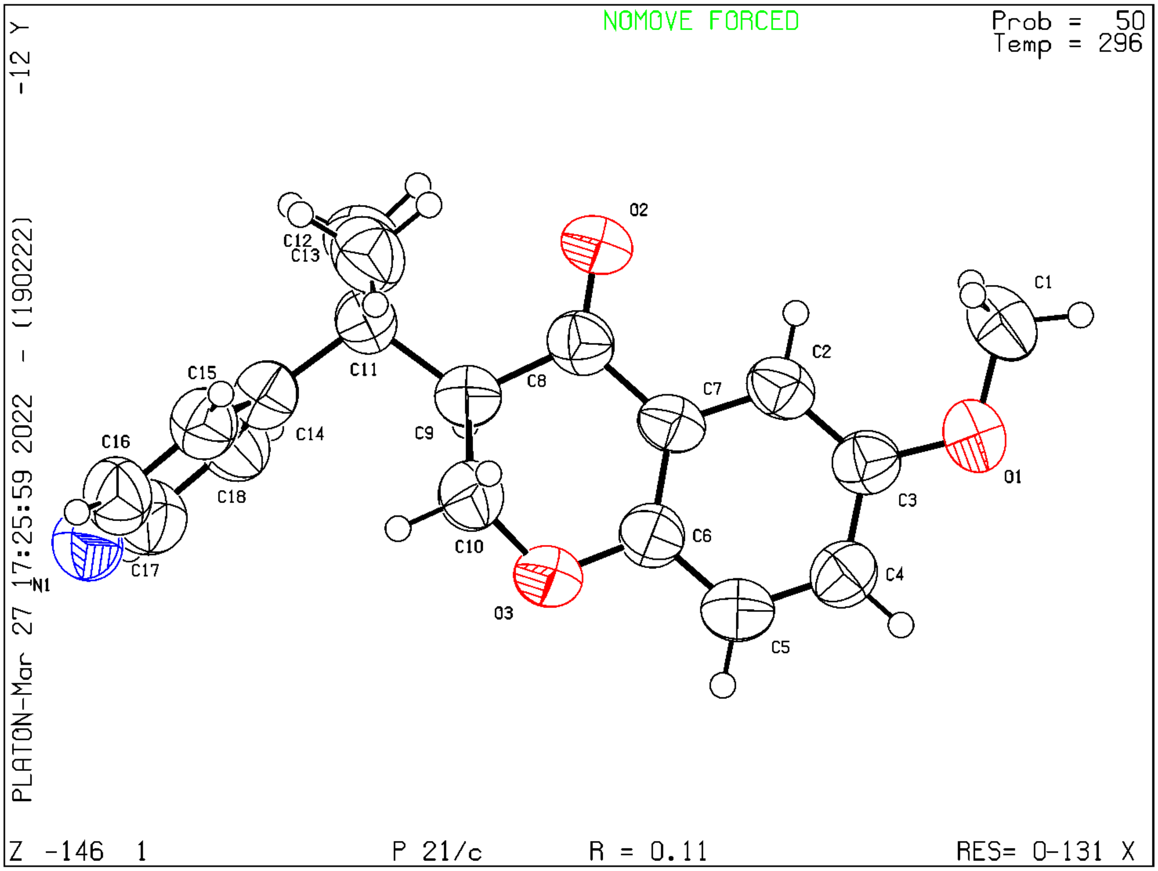

Supplement: Supplementary file 1 [file DataSheet2.PDF]
