# Supplementary material for: Redox-neutral and metal-free synthesis of 3-(arylmethyl)chroman-4-ones via visible-light-driven alkene acylarylation
Source: Front Chem. 2022 Oct 31;10:1059792. doi: 10.3389/fchem.2022.1059792 (PMC9660241; doi:10.3389/fchem.2022.1059792)
Supplement: Supplementary file 2 [file DataSheet3.DOCX]

Supplementary Material

**Contents**

[**1. General information S1**](#_Toc110622202)

[**2. The synthesis of starting materials S2**](#_Toc110622203)

[**3. Typical experimental procedure S4**](#_Toc110622204)

[**4. Characterization data of the products S4**](#_Toc110622205)

[**5. The application of the reaction S18**](#_Toc110622206)

[**6. Mechanistic studies S23**](#_Toc110622207)

[**7. X-ray crystal data S28**](#_Toc110622208)

[**8. Supplementary references S30**](#_Toc110622209)

[**9. Copies of NMR spectra for products S31**](#_Toc110622210)

1. **General information**

All reagents and starting materials, unless otherwise noted, were purchased from Energy, J&K, TCI, and Adamas-beta® Chemical company as reagent grade and used without further purification. Anhydrous solvents (including CH_2_Cl_2_, DCE, DMSO, MeCN, Water < 0.005%) were purchased from Energy, and used as received. Unless otherwise indicated, all syntheses and manipulations were carried out under argon atmosphere.

^1^H NMR, ^13^C NMR, and ^19^F NMR spectra were obtained with a Bruker AV II-400 spectrometer (^1^H: 400 MHz, ^13^C: 101 MHz, ^19^F: 376 MHz). The chemical shifts were measured with tetramethylsilane as the internal reference. The chemical shifts (*δ*) were expressed in ppm and *J* values were given in Hz. The following abbreviations were used to explain the multiplicities: s = singlet, d = doublet, t = triplet, q = quartet, m = multiplet, dd = doublet of doublets. TLC was performed using commercially prepared silica gel plates (GF254), and visualized under UV light 254 nm. Flash column chromatography was performed on silica gel (100–200 mesh). All mixed solvent eluents are reported as v/v solutions. Cyclic voltammetry tests were carried out with a CHI700E electrochemical workstation. Mass analysis data were acquired on a SCIEX UPLC (EXion)–QTOF (X500R). Melting points were measured using a Hanon MP470 apparatus.

1. **The synthesis of starting materials**
   1. **The preparation of** **alkenoic acids**

The alkenoic acids **1a-1v** were synthesized following the reported procedures.(De and Rigby, 2013; Carrillo-Arcos et al., 2016; Hoang et al., 2018; Chen et al., 2020)

- 1. **The preparation of photocatalysts**

Photocatalysts such as 3DPAFIPN, 3DPA2FBN, and 4CzIPN were synthesized according to literature report.(Speckmeier et al., 2018; Li et al., 2020) The spectral data of the photocatalysts are consistent with the literature data.

1. **Typical experimental procedure**

An oven-dried Schlenk tube (10 mL) equipped with a stirring bar was charged with P(*p*-tol)_3_ (0.6 mmol, 2.0 equiv.), 3DPAFIPN (0.006 mmol, 2 mol%), the corresponding cyanoarene (4.5 mmol, 1.5 equiv). The tube was connected to a vacuum line where it was evacuated and back-filled with Ar for three times. Then, alkenoic acid (0.3 mmol, 1.0 equiv.) and MeCN (6.0 mL) were added under Ar flow. The tube was placed approximately 2 cm from 30 W blue LEDs, and stirred at room temperature for 24 h. After completion, the mixture was concentrated in vacuo. The resulting residue was purified by flash column chromatography to afford the desired product.

1. **Characterization data of the products**

**3-(2-(pyridin-4-yl)propan-2-yl)chroman-4-one (3aa)**

**Physical State:** yellow oil.

**Yield:** 72%.

**^1^H NMR (400 MHz, CDCl_3_)** δ 8.62 – 8.50 (m, 2H), 7.87 (d, *J* = 7.8 Hz, 1H), 7.44 (t, *J* = 7.6 Hz, 1H), 7.33 – 7.27 (m, 2H), 7.01 (t, *J* = 7.3 Hz, 1H), 6.87 (d, *J* = 8.2 Hz, 1H), 4.37 – 4.30 (m, 1H), 4.23 (dd, *J* = 12.0, 5.3 Hz, 1H), 2.91 – 2.82 (m, 1H), 1.53 (s, 3H), 1.43 (s, 3H).

**^13^C NMR (101 MHz, CDCl_3_)** δ 192.5, 161.2, 156.4, 150.0, 136.0, 127.2, 122.0, 121.5, 121.3, 117.6, 68.9, 54.4, 39.8, 27.8, 24.0.

**HRMS** (ESI+)**:** calculated m/z [M+H]^+^ for [C_17_H_18_NO_2_]^+^: 268.1332, found: 268.1344.

**7-methyl-3-(2-(pyridin-4-yl)propan-2-yl)chroman-4-one (3ba)**

**Physical State:** yellow oil.

**Yield:** 65%.

**^1^H NMR (400 MHz, CDCl_3_)** δ 8.56 (d, *J* = 4.8 Hz, 2H), 7.76 (d, *J* = 8.0 Hz, 1H), 7.34 – 7.26 (m, 2H), 6.82 (d, *J* = 8.0 Hz, 1H), 6.67 (s, 1H), 4.30 (dd, *J* = 12.0, 4.0 Hz, 1H), 4.18 (dd, *J* = 12.0, 5.1 Hz, 1H), 2.86 – 2.77 (m, 1H), 2.33 (s, 3H), 1.52 (s, 3H), 1.42 (s, 3H).

**^13^C NMR (101 MHz, CDCl_3_)** δ 192.2, 161.2, 156.6, 149.9, 147.5, 127.1, 122.9, 121.4, 119.8, 117.6, 69.0, 54.4, 39.9, 27.9, 23.9, 21.9.

**HRMS** (ESI+): calculated m/z [M+H]^+^ for [C_18_H_20_NO_2_]^+^: 282.1489, found: 282.1496.

**7-methoxy-3-(2-(pyridin-4-yl)propan-2-yl)chroman-4-one (3ca)**

**Physical State:** white solid.

**Yield:** 48%.

**Melting Point:** 112.1-113.5 ℃

**^1^H NMR (400 MHz, CDCl_3_)** δ 8.55 (d, *J* = 4.5, 2H), 7.81 (d, *J* = 8.8, 1H), 7.29 (d, *J* = 4.7, 2H), 6.56 (d, *J* = 8.8, 1H), 6.29 (s, 1H), 4.30 (dd, *J* = 12.0, 4.0, 1H), 4.18 (dd, *J* = 12.0, 4.7, 1H), 3.81 (s, 3H), 2.80 – 2.72 (m, 1H), 1.51 (s, 3H), 1.41 (s, 3H).

**^13^C NMR (101 MHz, CDCl_3_)** δ 191.0, 166.0, 163.1, 156.6, 149.9, 128.9, 121.4, 115.9, 110.1, 100.4, 69.3, 55.6, 54.2, 40.0, 28.1, 23.9.

**HRMS** (ESI+): calculated m/z [M+H]^+^ for [C_18_H_20_NO_3_]^+^: 298.1438, found: 298.1432.

**7-chloro-3-(2-(pyridin-4-yl)propan-2-yl)chroman-4-one (3da)**

**Physical State:** yellow oil.

**Yield:** 54%.

**^1^H NMR (400 MHz, CDCl_3_)** δ 8.56 (d, *J* = 4.1, 2H), 7.80 (d, *J* = 8.4, 1H), 7.28 (d, *J* = 4.6, 2H), 6.98 (d, *J* = 8.4 Hz, 1H), 6.90 (s, 1H), 4.32 (dd, *J* = 12.1, 4.1, 1H), 4.21 (dd, *J* = 12.2, 5.3, 1H), 2.87 – 2.80 (m, 1H), 1.51 (s, 3H), 1.41 (s, 3H).

**^13^C NMR (101 MHz, CDCl_3_)** δ 191.4, 161.5, 156.1, 150.1, 141.8, 128.5, 122.4, 121.3, 120.5, 117.7, 69.3, 54.3, 39.9, 27.8, 23.9.

**HRMS** (ESI+): calculated m/z [M+H]^+^ for [C_17_H_17_ClNO_2_]^+^: 302.0942, found: 302.0955.

**7-fluoro-3-(2-(pyridin-4-yl)propan-2-yl)chroman-4-one (3ea)**

**Physical State:** yellow oil.

**Yield:** 60%.

**^1^H NMR (400 MHz, CDCl_3_)** δ 8.56 (d, *J* = 4.4, 2H), 7.96 – 7.82 (m, 1H), 7.29 (d, *J* = 5.7, 2H), 6.78 – 6.68 (m, 1H), 6.60 – 6.50 (m, 1H), 4.34 (dd, *J* = 12.1, 4.2, 1H), 4.24 (dd, *J* = 12.1, 5.2, 1H), 2.89 – 2.81 (m, 1H), 1.53 (s, 3H), 1.42 (s, 3H).

**^13^C NMR (101 MHz, CDCl_3_)** δ 191.1, 167.4 (d, *J* = 256.4), 162.8 (d, *J* = 13.6), 156.2, 150.0, 129.8 (d, *J* = 11.4), 121.3, 118.9 (d, *J* = 2.4), 110.0 (d, *J* = 22.7), 104.3 (d, *J* = 24.5), 69.5, 54.2, 39.9, 27.8, 24.0.

**^19^F NMR (376 MHz, CDCl_3_)** δ -100.36.

**HRMS** (ESI+): calculated m/z [M+H]^+^ for [C_17_H_17_FNO_2_]^+^: 286.1238, found: 286.1248.

**3-(2-(pyridin-4-yl)propan-2-yl)-7-(trifluoromethyl)chroman-4-one (3fa)**

**Physical State:** yellow solid.

**Yield:** 35%.

**^1^H NMR (400 MHz, CDCl_3_)** δ 8.58 (d, *J* = 4.2 Hz, 2H), 7.98 (d, *J* = 8.1 Hz, 1H), 7.30 (d, *J* = 4.7 Hz, 2H), 7.27 – 7.21 (m, 1H), 7.17 (s, 1H), 4.38 (dd, *J* = 12.2, 4.2 Hz, 1H), 4.27 (dd, *J* = 12.2, 5.7 Hz, 1H), 2.96 – 2.88 (m, 1H), 1.53 (s, 3H), 1.43 (s, 3H).

**^13^C NMR (101 MHz, CDCl_3_)** δ 191.6, 161.0, 156.0, 150.1, 137.0 (q, *J* = 32.9 Hz), 128.2, 124.0, 123.1 (q, *J* = 273.3 Hz), 121.2, 117.9 (q, *J* = 3.6 Hz), 115.3 (q, *J* = 3.9 Hz), 69.3, 54.4, 39.9, 27.7, 23.9.

**^19^F NMR (376 MHz, CDCl_3_)** δ -63.64.

**HRMS** (ESI+): calculated m/z [M+H]^+^ for [C_18_H_17_F_3_NO_2_]^+^: 336.1206, found: 336.1215.

**6-methoxy-3-(2-(pyridin-4-yl)propan-2-yl)chroman-4-one (3ga)**

**Physical State:** white solid.

**Yield:** 58%.

**Melting Point:** 80.6-82.6 ℃

**^1^H NMR (400 MHz, CDCl_3_)** δ 8.55 (d, *J* = 4.8, 2H), 7.33 – 7.27 (m, 3H), 7.05 (dd, *J* = 9.0, 2.6 Hz, 1H), 6.80 (d, *J* = 9.0 Hz, 1H), 4.29 (dd, *J* = 12.1, 4.2, 1H), 4.17 (dd, *J* = 12.0, 5.4, 1H), 3.78 (s, 3H), 2.85 – 2.79 (m, 1H), 1.51 (s, 3H), 1.41 (s, 3H).

**^13^C NMR (101 MHz, CDCl_3_)** δ 192.6, 156.5, 155.9, 154.1, 149.9, 125.2, 121.7, 121.3, 118.9, 107.4, 69.1, 55.8, 54.4, 39.9, 27.7, 24.1.

**HRMS** (ESI+): calculated m/z [M+H]^+^ for [C_18_H_20_NO_3_]^+^: 298.1438, found: 298.1445.

**6-bromo-3-(2-(pyridin-4-yl)propan-2-yl)chroman-4-one (3ha)**

**Physical State:** yellow oil.

**Yield:** 47%.

**^1^H NMR (400 MHz, CDCl_3_)** δ 8.62 – 8.49 (m, 2H), 7.98 (s, 1H), 7.52 (d, *J* = 8.8, 1H), 7.34 – 7.25 (m, 2H), 6.78 (d, *J* = 8.6, 1H), 4.37 – 4.28 (m, 1H), 4.27 – 4.19 (m, 1H), 2.89 – 2.79 (m, 1H), 1.52 (s, 3H), 1.42 (s, 3H).

**^13^C NMR (101 MHz, CDCl_3_)** δ 191.3, 160.1, 156.0, 150.0, 138.6, 129.6, 123.2, 121.3, 119.7, 114.2, 69.1, 54.2, 39.9, 27.8, 23.9.

**HRMS** (ESI+): calculated m/z [M+H]^+^ for [C_17_H_17_BrNO_2_]^+^: 346.0437, found: 346.0448.

**6-chloro-3-(2-(pyridin-4-yl)propan-2-yl)chroman-4-one (3ia)**

**Physical State:** colorless oil.

**Yield:** 49%.

**^1^H NMR (400 MHz, CDCl_3_)** δ 8.55 (d, *J* = 4.2, 2H), 7.87 – 7.79 (m, 1H), 7.41 – 7.34 (m, 1H), 7.30 – 7.26 (m, 2H), 6.82 (d, *J* = 8.8, 1H), 4.31 (dd, *J* = 12.1, 3.9, 1H), 4.22 (dd, *J* = 12.2, 5.2, 1H), 2.87 – 2.79 (m, 1H), 1.51 (s, 3H), 1.41 (s, 3H).

**^13^C NMR (101 MHz, CDCl_3_)** δ 191.4, 159.6, 156.0, 150.0, 135.8, 127.1, 126.5, 122.7, 121.3, 119.3, 69.1, 54.2, 39.9, 27.8, 23.9.

**HRMS** (ESI+): calculated m/z [M+H]^+^ for [C_17_H_17_ClNO_2_]^+^: 302.0942, found: 302.0954.

**5-fluoro-3-(2-(pyridin-4-yl)propan-2-yl)chroman-4-one (3ja)**

**Physical State:** yellow solid.

**Yield:** 24%.

**Melting Point:** 109.9-110.9 ℃

**^1^H NMR (400 MHz, CDCl_3_)** δ 8.55 (d, *J* = 4.5, 2H), 7.40 – 7.32 (m, 1H), 7.32 – 7.26 (m, 2H), 6.73 – 6.63 (m, 2H), 4.33 (dd, *J* = 12.0, 3.9, 1H), 4.24 (dd, *J* = 12.1, 5.7, 1H), 2.88 – 2.81 (m, 1H), 1.54 (s, 3H), 1.46 (s, 3H).

**^13^C NMR (101 MHz, CDCl_3_)** δ 190.3, 162.0 (d, *J* = 3.0), 161.7 (d, *J* = 265.8), 156.1, 149.9, 135.9 (d, *J* = 11.9), 121.3, 113.3 (d, *J* = 3.9), 112.0 (d, *J* = 8.6), 109.0 (d, *J* = 21.4), 68.7, 55.0, 39.8, 27.6, 24.1.

**^19^F NMR (376 MHz, CDCl_3_)** δ -111.01.

**HRMS** (ESI+): calculated m/z [M+H]^+^ for [C_17_H_17_FNO_2_]^+^: 286.1238, found: 286.1241.

**(phenyl(pyridin-4-yl)methyl)chroman-4-one (3ka)**

**Physical State:** yellow solid.

**Yield:** 72% (*dr* = 1.7:1).

**Melting Point:** 151.7-153.1 ℃

**^1^H NMR (400 MHz, CDCl_3_, major diastereomer)** δ 8.55 – 8.43 (m, 2H), 7.77 (d, *J* = 7.5 Hz, 1H), 7.50 (t, *J* = 7.3 Hz, 1H), 7.37 – 7.31 (m, 2H), 7.30 – 7.24 (m, 3H), 7.24 – 7.18 (m, 2H), 7.06 – 6.95 (m, 2H), 4.53 – 4.35 (m, 2H), 4.23 – 4.11 (m, 1H), 3.57 – 3.43 (m, 1H).

**^13^C NMR (101 MHz, CDCl_3_, major diastereomer)** δ 192.6, 161.0, 150.5, 149.8, 139.8, 136.1, 129.2, 128.4, 127.6, 123.4, 121.7, 120.4, 117.7, 69.4, 48.9, 48.1.

**HRMS** (ESI+): calculated m/z [M+H]^+^ for [C_21_H_18_NO_2_]^+^: 316.1332. found: 316.1339.

**^1^H NMR (400 MHz, CDCl_3_, minor diastereomer)** δ 8.59 – 8.52 (m, 2H), 7.77 (d, *J* = 7.9 Hz, 1H), 7.50 (t, *J* = 7.9 Hz, 1H), 7.35 – 7.17 (m, 7H), 7.08 – 6.95 (m, 2H), 4.62 – 4.44 (m, 2H), 4.22 – 4.12 (m, 1H), 3.56 – 3.44 (m, 1H).

**^13^C NMR (101 MHz, CDCl_3_, minor diastereomer)** δ 192.2, 160.9, 150.3, 139.5, 136.1, 128.7, 128.2, 127.7, 127.4, 123.7, 121.8, 120.6, 117.6, 69.2, 49.1, 47.8.

**HRMS** (ESI+): calculated m/z [M+H]^+^ for [C_21_H_18_NO_2_]^+^: 316.1332. found: 316.1341.

**3-(1-(pyridin-4-yl)ethyl)chroman-4-one (3la)**

**Physical State:** yellow oil.

**Yield:** 33% (*dr* = 2.0:1).

**^1^H NMR (400 MHz, CDCl_3_, diastereomer)** 8.59 – 8.55 (m, 4H), 8.55 – 8.50 (m, 2H), 7.91 (d, *J* = 7.8 Hz, 2H), 7.85 (d, *J* = 7.9 Hz, 1H), 7.58 – 7.42 (m, 3H), 7.22 (d, *J* = 5.0 Hz, 4H), 7.14 (d, *J* = 5.0 Hz, 2H), 7.08 – 6.99 (m, 3H), 6.99 – 6.91 (m, 3H), 4.51 – 4.38 (m, 2H), 4.36 – 4.27 (m, 2H), 4.03 – 3.95 (m, 2H), 3.66 – 3.55 (m, 1H), 3.35 – 3.21 (m, 2H), 2.96 – 2.86 (m, 1H), 2.67 – 2.59 (m, 2H), 1.36 (d, *J* = 7.2 Hz, 3H), 1.32 (d, *J* = 7.1 Hz, 6H).

**^13^C NMR (101 MHz, CDCl_3_, diastereomer)** δ 193.1, 192.5, 161.3, 161.1, 153.0, 152.5, 150.1, 149.8, 136.1, 136.1, 127.6, 127.4, 123.1, 122.9, 121.7, 121.6, 121.0, 120.3, 117.7, 117.7, 68.7, 67.8, 51.9, 51.4, 36.3, 36.1, 19.0, 15.6.

**HRMS** (ESI+): calculated m/z [M+H]^+^ for [C_16_H_16_NO_2_]^+^: 254.1176, found: 254.1186.

**3-phenyl-3-(pyridin-4-ylmethyl)chroman-4-one (3ma)**

**Physical State:** yellow oil.

**Yield:** 22%.

**^1^H NMR (400 MHz, CDCl_3_)** δ 8.66 – 8.47 (m, 2H), 7.88 (d, *J* = 5.9, 1H), 7.49 – 7.31 (m, 7H), 7.30 – 7.22 (m, 1H), 7.20 – 7.05 (m, 2H), 4.74 (d, *J* = 12.8, 1H), 4.58 (d, *J* = 12.8, 1H), 3.58 (d, *J* = 12.6, 1H), 3.49 (d, *J* = 12.6, 1H).

**^13^C NMR (101 MHz, CDCl_3_)** δ 196.9, 164.0, 154.9, 150.2, 143.9, 134.3, 130.0, 128.9, 127.5, 127.4, 123.0, 122.5, 120.3, 82.1, 54.4, 53.2.

**HRMS** (ESI+): calculated m/z [M+H]^+^ for [C_21_H_18_NO_2_]^+^: 316.1332. found: 316.1344.

**3-methyl-3-(pyridin-4-ylmethyl)chroman-4-one (3na)**

**Physical State:** yellow oil.

**Yield:** 20%.

**^1^H NMR (400 MHz, CDCl_3_)** δ 8.93 – 8.50 (m, 2H), 7.91 (d, *J* = 7.4 Hz, 1H), 7.79 – 7.63 (m, 2H), 7.53 (t, *J* = 6.8 Hz, 1H), 7.10 (t, *J* = 6.9 Hz, 1H), 6.99 (d, *J* = 7.9 Hz, 1H), 4.28 – 4.08 (m, 2H), 3.32 – 3.20 (m, 1H), 3.13 – 3.02 (m, 1H), 1.20 (s, 3H).

**^13^C NMR (101 MHz, CDCl_3_)** δ 195.9, 161.1, 149.7, 145.1, 136.0, 127.9, 125.9, 121.9, 119.6, 117.8, 73.6, 45.4, 38.9, 17.3.

**HRMS** (ESI+): calculated m/z [M+H]^+^ for [C_16_H_16_NO_2_]^+^: 254.1176, found: 254.1186.

**3-(pyridin-4-ylmethyl)chroman-4-one (3oa)**

**Physical State:** white solid.

**Yield:** 25%.

**Melting Point:** 75.1-77.4 ℃

**^1^H NMR (400 MHz, CDCl_3_)** δ 8.55 (d, *J* = 4.3, 2H), 7.92 (d, *J* = 7.8, 1H), 7.50 (t, *J* = 7.7, 1H), 7.19 (d, *J* = 4.8, 2H), 7.05 (t, *J* = 7.5, 1H), 6.97 (d, *J* = 8.3, 1H), 4.39 (dd, *J* = 11.5, 4.4, 1H), 4.21 – 4.10 (m, 1H), 3.33 – 3.24 (m, 1H), 3.06 – 2.94 (m, 1H), 2.77 – 2.67 (m, 1H).

**^13^C NMR (101 MHz, CDCl_3_)** δ 192.9, 161.5, 150.0, 147.6, 136.2, 127.5, 124.4, 121.7, 120.4, 117.9, 69.4, 46.7, 31.7.

**HRMS** (ESI+): calculated m/z [M+H]^+^ for [C_15_H_14_NO_2_]^+^: 240.1019, found: 240.1018.

**3-(2-(pyridin-4-yl)propan-2-yl)thiochroman-4-one (3pa)**

**Physical State:** yellow oil.

**Yield:** 52%.

**^1^H NMR (400 MHz, CDCl_3_)** δ 8.55 (d, *J* = 4.5, 2H), 7.96 (d, *J* = 7.9, 1H), 7.38 – 7.32 (m, 1H), 7.31 (d, *J* = 4.9, 2H), 7.20 (d, *J* = 7.9, 1H), 7.14 (t, *J* = 7.5, 1H), 3.37 – 3.29 (m, 1H), 3.21 – 3.12 (m, 1H), 2.99 – 2.91 (m, 1H), 1.55 (s, 3H), 1.47 (s, 3H).

**^13^C NMR (101 MHz, CDCl_3_)** δ 194.3, 158.0, 149.9, 141.1, 133.0, 132.2, 129.5, 127.1, 124.9, 121.0, 55.9, 40.5, 28.3, 25.6, 25.0.

**HRMS** (ESI+): calculated m/z [M+H]^+^ for [C_17_H_18_NOS]^+^: 284.1104, found: 284.1115.

**1-butyryl-3-(2-(pyridin-4-yl)propan-2-yl)-2,3-dihydroquinolin-4(1*H*)-one (3qa)**

**Physical State:** yellow oil.

**Yield:** 60%.

**^1^H NMR (400 MHz, CDCl_3_)** δ 8.57 (d, *J* = 4.0, 2H), 7.94 (d, *J* = 7.7, 1H), 7.51 (t, *J* = 7.5, 1H), 7.48 – 7.39 (m, 1H), 7.31 – 7.26 (m, 2H), 7.26 – 7.20 (m, 1H), 4.60 – 4.26 (m, 1H), 3.66 – 3.54 (m, 1H), 3.08 (dd, *J* = 10.5, 4.1, 1H), 2.37 – 2.22 (m, 2H), 1.67 (m, 2H), 1.56 (s, 3H), 1.43 (s, 3H), 0.92 (t, *J* = 7.3, 3H).

**^13^C NMR (101 MHz, CDCl_3_)** δ 195.1, 172.2, 157.1, 150.0, 143.4, 133.9, 127.9, 127.0, 125.4, 123.5, 121.1, 56.9, 46.0, 40.1, 36.4, 26.7, 25.1, 18.9, 13.8.

**HRMS** (ESI+): calculated m/z [M+H]^+^ for [C_21_H_25_N_2_O_2_]^+^: 337.1911, found: 337.1917.

**2-(2-(pyridin-4-yl)propan-2-yl)-3,4-dihydronaphthalen-1(2*H*)-one (3ra)**

**Physical State:** yellow solid.

**Yield:** 60%.

**Melting Point:** 108.4-109.4 ℃

**^1^H NMR (400 MHz, CDCl_3_)** δ 8.68 – 8.42 (m, 2H), 7.89 (d, *J* = 7.6, 1H), 7.44 (t, *J* = 7.2, 1H), 7.35 – 7.24 (m, 3H), 7.20 (d, *J* = 7.4, 1H), 3.11 – 2.88 (m, 3H), 2.13 – 2.02 (m, 1H), 2.01 – 1.85 (m, 1H), 1.55 (s, 3H), 1.39 (s, 3H).

**^13^C NMR (101 MHz, CDCl_3_)** δ 198.2, 159.3, 149.7, 143.2, 133.8, 133.1, 128.5, 127.3, 126.6, 121.1, 56.6, 40.0, 29.9, 25.7, 25.7, 25.1.

**HRMS** (ESI+): calculated m/z [M+H]^+^ for [C_18_H_20_NO]^+^: 266.1539, found: 266.1537.

**2-(2-(pyridin-4-yl)propan-2-yl)-2,3-dihydro-1*H*-pyrrolo[1,2-*a*]indol-1-one (3sa)**

**Physical State:** yellow solid.

**Yield:** 42%.

**Melting Point:** 205.4-206.3 ℃

**^1^H NMR (400 MHz, CDCl_3_)** δ 8.60 (d, *J* = 5.2 Hz, 2H), 7.74 (d, *J* = 8.2 Hz, 1H), 7.35 – 7.27 (m, 4H), 7.20 – 7.14 (m, 1H), 6.99 (s, 1H), 4.22 (dd, *J* = 11.5, 8.2 Hz, 1H), 3.80 (dd, *J* = 11.5, 4.5 Hz, 1H), 3.59 (dd, *J* = 8.1, 4.5 Hz, 1H), 1.66 (s, 3H), 1.39 (s, 3H).

**^13^C NMR (101 MHz, CDCl_3_)** δ 193.5, 156.4, 150.2, 136.3, 135.0, 132.0, 125.2, 124.2, 121.6, 121.1, 110.5, 99.2, 58.5, 44.0, 41.1, 28.0, 21.8.

**HRMS** (ESI+): calculated m/z [M+H]^+^ for [C_19_H_19_N_2_O]^+^: 291.1492, found: 291.1498.

**8-(2-(pyridin-4-yl)propan-2-yl)-7,8-dihydropyrido[1,2-*a*]indol-9(6*H*)-one (3ta)**

**Physical State:** yellow solid.

**Yield:** 54%.

**Melting Point:** 200.5-201.7 ℃

**^1^H NMR (400 MHz, CDCl_3_)** δ 8.56 (d, *J* = 5.3 Hz, 2H), 7.70 (d, *J* = 8.1 Hz, 1H), 7.39 – 7.27 (m, 4H), 7.22 (s, 1H), 7.15 (t, *J* = 7.4 Hz, 1H), 4.41 – 4.28 (m, 1H), 4.09 – 3.95 (m, 1H), 3.14 – 2.99 (m, 1H), 2.25 – 2.14 (m, 2H), 1.63 (s, 3H), 1.45 (s, 3H).

**^13^C NMR (101 MHz, CDCl_3_)** δ 190.1, 158.5, 149.9, 136.9, 134.5, 126.9, 125.6, 123.4, 121.2, 121.1, 110.2, 105.7, 54.9, 41.6, 40.2, 26.4, 25.8, 24.8.

**HRMS** (ESI+): calculated m/z [M+H]^+^ for [C_20_H_21_N_2_O]^+^: 305.1648, found: 305.1657.

**3-(2-(2-(4-(*tert*-butyl)phenyl)pyridin-4-yl)propan-2-yl)chroman-4-one (3ab)**

**Physical State:** yellow oil.

**Yield:** 63%.

**^1^H NMR (400 MHz, CDCl_3_)** δ 8.62 (d, *J* = 4.5 Hz, 1H), 7.95 – 7.83 (m, 3H), 7.71 (s, 1H), 7.50 (d, *J* = 8.1 Hz, 2H), 7.41 (t, *J* = 7.7 Hz, 1H), 7.24 (d, *J* = 4.1 Hz, 1H), 6.98 (t, *J* = 7.5 Hz, 1H), 6.85 (d, *J* = 8.3 Hz, 1H), 4.34 (dd, *J* = 12.1, 4.2 Hz, 1H), 4.26 (dd, *J* = 12.1, 5.3 Hz, 1H), 2.92 – 2.86 (m, 1H), 1.57 (s, 3H), 1.47 (s, 3H), 1.36 (s, 9H).

**^13^C NMR (101 MHz, CDCl_3_)** δ 192.5, 161.2, 157.6, 157.5, 152.4, 149.3, 136.3, 136.0, 127.2, 126.9, 125.8, 122.0, 121.5, 119.7, 118.4, 117.6, 69.0, 54.5, 40.1, 34.7, 31.3, 27.9, 24.1.

**HRMS** (ESI+): calculated m/z [M+H]^+^ for [C_27_H_30_NO_2_]^+^: 400.2271, found: 400.2275.

**3-(2-(2-(*p*-tolyl)pyridin-4-yl)propan-2-yl)chroman-4-one (3ac)**

**Physical State:** colorless oil.

**Yield:** 71%.

**^1^H NMR (400 MHz, CDCl_3_)** δ 8.61 (d, *J* = 5.0 Hz, 1H), 7.92 – 7.82 (m, 3H), 7.69 (s, 1H), 7.43 (t, *J* = 7.6 Hz, 1H), 7.31 – 7.26 (m, 2H), 7.23 (d, *J* = 4.6 Hz, 1H), 7.00 (t, *J* = 7.4 Hz, 1H), 6.86 (d, *J* = 8.3 Hz, 1H), 4.38 – 4.32 (m, 1H), 4.31 – 4.24 (m, 1H), 2.94 – 2.83 (m, 1H), 2.41 (s, 3H), 1.58 (s, 3H), 1.48 (s, 3H).

**^13^C NMR (101 MHz, CDCl_3_)** δ 192.7, 161.2, 157.8, 157.0, 149.8, 139.0, 136.8, 136.0, 129.5, 127.2, 126.9, 122.0, 121.5, 119.6, 118.0, 117.6, 69.0, 54.5, 40.1, 28.2, 23.9, 21.3.

**HRMS** (ESI+): calculated m/z [M+H]^+^ for [C_24_H_24_NO_2_]^+^: 358.1802, found: 358.1809.

**3-(2-(2-(4-methoxyphenyl)pyridin-4-yl)propan-2-yl)chroman-4-one (3ad)**

**Physical State:** yellow oil.

**Yield:** 57%.

**^1^H NMR (400 MHz, CDCl_3_)** δ 8.58 (d, *J* = 5.3 Hz, 1H), 7.91 (d, *J* = 8.7 Hz, 2H), 7.89 – 7.86 (m, 1H), 7.65 (s, 1H), 7.44 – 7.38 (m, 1H), 7.20 – 7.17 (m, 1H), 7.02 – 6.96 (m, 3H), 6.85 (d, *J* = 8.3 Hz, 1H), 4.34 (dd, *J* = 12.1, 4.4 Hz, 1H), 4.27 (dd, *J* = 12.2, 5.2 Hz, 1H), 3.86 (s, 3H), 2.91 – 2.85 (m, 1H), 1.57 (s, 3H), 1.47 (s, 3H).

**^13^C NMR (101 MHz, CDCl_3_)** δ 192.7, 161.2, 160.5, 157.5, 156.9, 149.7, 135.9, 132.2, 128.3, 127.2, 122.0, 121.5, 119.2, 117.6, 117.5, 114.1, 69.0, 55.4, 54.5, 40.1, 28.1, 23.9.

**HRMS** (ESI+): calculated m/z [M+H]^+^ for [C_24_H_24_NO_3_]^+^: 374.1751, found: 374.1764.

**3-(2-(2-(4-fluorophenyl)pyridin-4-yl)propan-2-yl)chroman-4-one (3ae)**

**Physical State:** yellow oil

**Yield:** 72%.

**^1^H NMR (400 MHz, CDCl_3_)** δ 8.60 (d, *J* = 5.3 Hz, 1H), 7.98 – 7.88 (m, 2H), 7.87 (dd, *J* = 7.9, 1.7 Hz, 1H), 7.65 (d, *J* = 1.3 Hz, 1H), 7.46 – 7.37 (m, 1H), 7.24 (dd, *J* = 5.3, 1.8 Hz, 1H), 7.18 – 7.11 (m, 2H), 7.02 – 6.95 (m, 1H), 6.87 – 6.82 (m, 1H), 4.36 (dd, *J* = 12.1, 4.4, 1H), 4.29 (dd, *J* = 12.1, 5.3, 1H), 2.92 – 2.87 (m, 1H), 1.58 (s, 3H), 1.48 (s, 3H).

**^13^C NMR (101 MHz, CDCl_3_)** δ 192.5, 163.5 (d, *J* = 248.4), 161.2, 157.2, 156.8, 149.8, 136.0, 135.7 (d, *J* = 3.1), 128.9 (d, *J* = 8.3), 127.2, 122.0, 121.5, 119.8, 118.0, 117.6, 115.6 (d, *J* = 21.6), 68.9, 54.6, 40.1, 27.8, 24.3.

**^19^F NMR (376 MHz, CDCl_3_)** δ -113.06.

**HRMS** (ESI+): calculated m/z [M+H]^+^ for [C_23_H_21_FNO_2_]^+^: 362.1551, found: 362.1560.

**3-(2-(2-(*tert*-butyl)pyridin-4-yl)propan-2-yl)chroman-4-one (3af)**

**Physical State:** white solid.

**Yield:** 58%.

**Melting Point:** 61.1-61.0 ℃

**^1^H NMR (400 MHz, CDCl_3_)** δ 8.49 (d, *J* = 5.2, 1H), 7.87 (d, *J* = 7.9, 1H), 7.42 (t, *J* = 7.7, 1H), 7.30 (s, 1H), 7.10 (d, *J* = 5.1, 1H), 6.99 (t, *J* = 7.5, 1H), 6.83 (d, *J* = 8.3, 1H), 4.29 (dd, *J* = 12.1, 4.2, 1H), 4.24 (dd, *J* = 12.2, 4.9, 1H), 2.83 – 2.75 (m, 1H), 1.53 (s, 3H), 1.43 (s, 3H), 1.34 (s, 9H).

**^13^C NMR (101 MHz, CDCl_3_)** δ 192.8, 169.4, 161.2, 156.1, 148.7, 135.9, 127.2, 122.0, 121.4, 118.4, 117.6, 116.5, 69.0, 54.7, 40.1, 37.5, 30.3, 28.3, 24.0.

**HRMS** (ESI+): calculated m/z [M+H]^+^ for [C_21_H_26_NO_2_]^+^: 324.1958, found: 324.1971.

**3-(2-(2-fluoropyridin-4-yl)propan-2-yl)chroman-4-one (3ag)**

**Physical State:** yellow oil.

**Yield:** 22%.

**^1^H NMR (400 MHz, CDCl_3_)** δ 8.15 (d, *J* = 5.2, 1H), 7.85 (d, *J* = 7.8, 1H), 7.45 (t, *J* = 7.5, 1H), 7.17 (d, *J* = 4.7, 1H), 7.01 (t, *J* = 7.4, 1H), 6.92 (s, 1H), 6.88 (d, *J* = 8.3, 1H), 4.38 (dd, *J* = 12.1, 4.0, 1H), 4.26 (dd, *J* = 12.0, 5.8, 1H), 2.91 – 2.83 (m, 1H), 1.52 (s, 3H), 1.42 (s, 3H).

**^13^C NMR (101 MHz, CDCl_3_)** δ 192.1, 164.4 (d, *J* = 238.2), 162.9 (d, *J* = 7.5), 161.1, 147.6 (d, *J* = 15.2), 136.1, 127.2, 121.8, 121.6, 119.1 (d, *J* = 3.8), 117.6, 107.2 (d, *J* = 37.8), 68.8, 54.4, 40.1 (d, *J* = 2.5), 27.2, 24.6.

**^19^F NMR (376 MHz, CDCl_3_)** δ -67.92.

**HRMS** (ESI+): calculated m/z [M+H]^+^ for [C_17_H_17_FNO_2_]^+^: 286.1238, found: 286.1251.

**3-(2-(3-chloropyridin-4-yl)propan-2-yl)chroman-4-one (3ah)**

**Physical State:** yellow solid.

**Yield:** 17%.

**Melting Point:** 120.4-121.5 ℃

**^1^H NMR (400 MHz, CDCl_3_)** δ 8.54 (s, 1H), 8.48 (d, *J* = 4.3, 1H), 7.85 (d, *J* = 7.8, 1H), 7.46 (t, *J* = 7.7, 1H), 7.38 (d, *J* = 4.5, 1H), 7.01 (t, *J* = 7.4, 1H), 6.92 (d, *J* = 8.3, 1H), 4.48 – 4.40 (m, 1H), 4.29 – 4.20 (m, 1H), 4.06 – 3.97 (m, 1H), 1.64 (s, 3H), 1.51 (s, 3H).

**^13^C NMR (101 MHz, CDCl_3_)** δ 192.7, 161.4, 152.7, 151.6, 148.3, 135.9, 130.6, 127.3, 123.2, 122.1, 121.6, 117.7, 69.1, 49.4, 40.9, 25.1, 24.5.

**HRMS** (ESI+): calculated m/z [M+H]^+^ for [C_17_H_17_ClNO_2_]^+^ : 302.0942, found: 302.0953.

**3-(2-(3-chloro-5-(trifluoromethyl)pyridin-2-yl)propan-2-yl)chroman-4-one (3ai)**

**Physical State:** white solid.

**Yield:** 38%.

**Melting Point:** 112.9-113.8 ℃

**^1^H NMR (400 MHz, CDCl_3_)** δ 8.67 (s, 1H), 7.87 (s, 1H), 7.82 (d, *J* = 7.8 Hz, 1H), 7.46 (t, *J* = 7.7 Hz, 1H), 7.00 (t, *J* = 7.5 Hz, 1H), 6.95 (d, *J* = 8.3 Hz, 1H), 4.63 – 4.51 (m, 2H), 3.98 – 3.90 (m, 1H), 1.68 (s, 3H), 1.64 (s, 3H).

**^13^C NMR (101 MHz, CDCl_3_)** δ 192.9, 165.3, 161.4, 142.5 (q, *J* = 4.0 Hz), 136.4 (q, *J* = 3.5 Hz), 135.6, 130.2, 127.4, 125.5 (q, *J* = 33.4 Hz), 122.7 (q, *J* = 272.7 Hz), 122.1, 121.5, 117.6, 69.0, 52.2, 44.5, 25.2, 24.2.

**^19^F NMR (376 MHz, CDCl_3_)** δ -62.23.

**HRMS** (ESI+): calculated m/z [M+H]^+^ for [C_18_H_16_ClF_3_NO_2_]^+^: 370.0816, found: 370.0821.

**4-(2-(4-oxochroman-3-yl)propan-2-yl)picolinonitrile (3aj)**

**Physical State:** white solid.

**Yield:** 38%.

**Melting Point:** 100.0-101.7 ℃

**^1^H NMR (400 MHz, CDCl_3_)** δ 8.63 (d, *J* = 4.7 Hz, 1H), 7.76 (d, *J* = 7.8 Hz, 1H), 7.60 (s, 1H), 7.43 (t, *J* = 7.7 Hz, 1H), 7.32 (d, *J* = 4.7 Hz, 1H), 6.97 (t, *J* = 7.5 Hz, 1H), 6.90 (d, *J* = 8.3 Hz, 1H), 4.62 (dd, *J* = 11.5, 4.6 Hz, 1H), 4.54 – 4.45 (m, 1H), 3.68 – 3.58 (m, 1H), 1.50 (s, 3H), 1.47 (s, 3H).

**^13^C NMR (101 MHz, CDCl_3_)** δ 193.0, 168.8, 161.4, 149.4, 135.7, 127.1, 122.4, 121.8, 121.5, 121.4, 120.6, 117.5, 117.0, 69.0, 53.7, 42.0, 27.0, 24.4.

**HRMS** (ESI+): calculated m/z [M+H]^+^ for [C_18_H_17_N_2_O_2_]^+^: 293.1285, found: 293.1294.

**2-(2-(4-oxochroman-3-yl)propan-2-yl)isonicotinonitrile (3aj')**

**Physical State:** yellow oil.

**Yield:** 16%.

**^1^H NMR (400 MHz, CDCl_3_)** δ 8.59 (d, *J* = 5.0 Hz, 1H), 7.80 (d, *J* = 7.6 Hz, 1H), 7.68 (s, 1H), 7.51 – 7.40 (m, 2H), 7.00 (t, *J* = 7.3 Hz, 1H), 6.85 (d, *J* = 8.3 Hz, 1H), 4.51 – 4.43 (m, 1H), 4.36 (dd, *J* = 12.0, 6.2 Hz, 1H), 2.95 – 2.86 (m, 1H), 1.54 (s, 3H), 1.46 (s, 3H).

**^13^C NMR (101 MHz, CDCl_3_)** δ 191.7, 161.0, 158.4, 150.9, 136.3, 134.0, 127.2, 126.4, 124.6, 121.8, 121.6, 117.6, 117.4, 68.5, 54.4, 39.6, 26.0, 25.7.

**HRMS** (ESI+): calculated m/z [M+H]^+^ for [C_18_H_17_N_2_O_2_]^+^: 293.1285, found: 293.1299.

**3-(2-(quinolin-2-yl)propan-2-yl)chroman-4-one (3ak)**

**Physical State:** yellow oil.

**Yield:** 49%.

**^1^H NMR (400 MHz, CDCl_3_)** δ 8.13 (d, *J* = 8.6 Hz, 1H), 7.92 (d, *J* = 8.4 Hz, 1H), 7.84 (d, *J* = 7.3 Hz, 1H), 7.78 (d, *J* = 8.0 Hz, 1H), 7.63 (t, *J* = 7.5 Hz, 1H), 7.57 (d, *J* = 8.6 Hz, 1H), 7.47 (t, *J* = 7.4 Hz, 1H), 7.41 (t, *J* = 7.3 Hz, 1H), 6.97 (t, *J* = 7.4 Hz, 1H), 6.90 (d, *J* = 8.3 Hz, 1H), 4.64 – 4.56 (m, 2H), 3.78 – 3.68 (m, 1H), 1.62 (s, 3H), 1.61 (s, 3H).

**^13^C NMR (101 MHz, CDCl_3_)** δ 193.7, 166.5, 161.5, 147.3, 136.3, 135.4, 129.3, 129.1, 127.3, 127.2, 126.5, 125.9, 122.4, 121.2, 118.1, 117.5, 69.5, 54.2, 42.8, 26.6, 26.3.

**HRMS** (ESI+): calculated m/z [M+H]^+^ for [C_21_H_20_NO_2_]^+^: 318.1489, found: 318.1496.

**3-(2-(isoquinolin-1-yl)propan-2-yl)chroman-4-one (3al)**

**Physical State:** yellow oil.

**Yield:** 48%.

**^1^H NMR (400 MHz, CDCl_3_)** δ 8.52 (d, *J* = 8.6 Hz, 1H), 8.44 – 8.35 (m, 1H), 7.92 (d, *J* = 7.7 Hz, 1H), 7.86 (d, *J* = 8.1 Hz, 1H), 7.64 (t, *J* = 7.2 Hz, 1H), 7.60 – 7.50 (m, 2H), 7.44 (t, *J* = 7.6 Hz, 1H), 7.02 (t, *J* = 7.4 Hz, 1H), 6.91 (d, *J* = 8.3 Hz, 1H), 4.52 – 4.43 (m, 1H), 4.40 – 4.31 (m, 1H), 3.75 – 3.65 (m, 1H), 1.92 – 1.82 (m, 6H).

**^13^C NMR (101 MHz, CDCl_3_)** δ 193.3, 163.7, 161.3, 140.2, 137.7, 135.3, 129.1, 128.7, 127.3, 126.4, 126.3, 126.0, 122.7, 121.3, 120.4, 117.6, 69.4, 54.5, 45.9, 28.2, 27.2.

**HRMS** (ESI+): calculated m/z [M+H]^+^ for [C_21_H_20_NO_2_]^+^: 318.1489, found: 318.1500.

**4-(2-(7-methoxy-4-oxochroman-3-yl)propan-2-yl)benzonitrile (3cm)**

**Physical State:** yellow solid.

**Yield:** 63%.

**Melting Point:** 117.7-119.1 ℃

**^1^H NMR (400 MHz, CDCl_3_)** δ 7.77 (d, *J* = 8.9 Hz, 1H), 7.60 (d, *J* = 8.6 Hz, 2H), 7.49 (d, *J* = 8.6 Hz, 2H), 6.54 (dd, *J* = 8.9, 2.4 Hz, 1H), 6.26 (d, *J* = 2.4 Hz, 1H), 4.30 (dd, *J* = 12.1, 4.4 Hz, 1H), 4.18 (dd, *J* = 12.1, 4.9 Hz, 1H), 3.80 (s, 3H), 2.79 – 2.72 (m, 1H), 1.52 (s, 3H), 1.42 (s, 3H).

**^13^C NMR (101 MHz, CDCl_3_)** δ 191.0, 166.0, 163.1, 153.1, 132.1, 128.9, 127.0, 118.9, 115.9, 110.1, 100.4, 69.3, 55.7, 54.5, 40.5, 28.0, 24.8.

**HRMS** (ESI+): calculated m/z [M+H]^+^ for [C_20_H_20_NO_3_]^+^: 322.1438, found: 322.1452.

1. **The application of the reaction**
   1. **The synthesis of 3-(pyridylmethyl)chromone (4)**

A 10 mL tube with a magnetic stir bar was charged with 3-(2-(pyridin-4-yl)propan-2-yl)chroman-4-one (**3aa**) (0.2 mmol, 1.0 equiv.), iodine (0.02 mmol, 0.1 equiv.), and DMSO (2 mL). The resulting mixture was refluxed for 2 h. After cooling, the reaction mixture was poured into ice and water. A small amount of Na_2_S_2_O_3_ was added and the reaction miture stirred for some minutes. Then extracted with ether. The combined organic layers were washed with brine, dried over anhydrous Na_2_SO_4_, filtered, then concentrated in vacuo, and purified by column chromatography (PE/EA = 3/1) to afford the desired product (**4**) in 86% yield.

**3-(2-(pyridin-4-yl)propan-2-yl)-4*H*-chromen-4-one** **(4)**

**Physical State:** yellow oil.

**Yield:** 86%.

**Melting Point:** 160.7-161.7 ℃

**^1^H NMR (400 MHz, CDCl_3_)** δ 8.49 (d, *J* = 4.3 Hz, 2H), 8.06 (dd, *J* = 8.0, 1.5 Hz, 1H), 8.02 (s, 1H), 7.71 – 7.60 (m, 1H), 7.46 (d, *J* = 8.3 Hz, 1H), 7.39 – 7.30 (m, 1H), 7.24 (d, *J* = 5.9 Hz, 2H), 1.67 (s, 6H).

**^13^C NMR (101 MHz, CDCl_3_)** δ 176.2, 157.7, 156.1, 152.4, 149.3, 133.6, 130.1, 126.1, 125.1, 124.4, 120.8, 117.9, 39.8, 27.7.

**HRMS** (ESI+): calculated m/z [M+H]^+^ for [C_17_H_16_NO_2_]^+^: 266.1176, found: 266.1185.

- 1. **The synthesis of 3-benzylchromone (5)**

A 10 mL tube with a magnetic stir bar was charged with 4-(2-(7-methoxy-4-oxochroman-3-yl)propan-2-yl)benzonitrile (**3cm**) (0.2 mmol, 1.0 equiv.), iodine (0.02 mmol, 0.1 equiv.), and DMSO (2 mL). The resulting mixture was refluxed for 2 h. After cooling, the reaction mixture was poured into ice and water. A small amount of Na_2_S_2_O_3_ was added and the reaction miture stirred for some minutes. Then extracted with ether. The combined organic layers were washed with brine, dried over anhydrous Na_2_SO_4_, filtered, then concentrated in vacuo, and purified by column chromatography (PE/EA = 3/1) to afford the desired product (**5**) in 75% yield.

**4-(2-(7-methoxy-4-oxo-4*H*-chromen-3-yl)propan-2-yl)benzonitrile (5)**

**Physical State:** white solid.

**Yield:** 75%.

**Melting Point:** 193.8-194.8 ℃

**^1^H NMR (400 MHz, CDCl_3_)** δ 7.99 – 7.93 (m, 2H), 7.56 (d, *J* = 8.5 Hz, 2H), 7.42 (d, *J* = 8.5 Hz, 2H), 6.91 (dd, *J* = 8.9, 2.4 Hz, 1H), 6.83 (d, *J* = 2.3 Hz, 1H), 3.90 (s, 3H), 1.67 (s, 6H).

**^13^C NMR (101 MHz, CDCl_3_)** δ 175.6, 164.0, 157.9, 154.0, 151.9, 132.1, 130.3, 127.5, 126.1, 119.2, 118.4, 114.5, 109.5, 99.9, 55.9, 40.2, 28.1.

**HRMS** (ESI+): calculated m/z [M+H]^+^ for [C_20_H_18_NO_3_]^+^: 320.1281, found: 320.1288.

- 1. **The synthesis of 3-benzylchroman-4-one-based amide (6)**

To a stirred solution of 4-(2-(7-methoxy-4-oxochroman-3-yl)propan-2-yl)benzonitrile (**3cm**) (0.2 mmol, 1.0 equiv.) in DMSO (2 mL), cooled in an ice bath, was added 30% H_2_O_2_ (120 μL) and K_2_CO_3_ (4 mg), the reaction was allowed to warm up to temperature. After 24 h, the completion of the reaction was monitored by TLC, the mixture was quenched with water, and then extracted with ether. The combined organic layers were washed with brine, dried over anhydrous Na_2_SO_4_, filtered, then concentrated in vacuo, and purified by column chromatography (DCM/MeOH = 30/1) to afford the desired product **6** in 92% yield.

**4-(2-(7-methoxy-4-oxochroman-3-yl)propan-2-yl)benzamide (6)**

**Physical State:** white solid.

**Yield:** 92%.

**Melting Point:** 159.5-160.4 ℃

**^1^H NMR (400 MHz, CDCl_3_)** δ 7.85 – 7.76 (m, 3H), 7.45 (d, *J* = 8.2 Hz, 2H), 6.54 (dd, *J* = 8.8, 2.2 Hz, 1H), 6.46 – 6.33 (m, 2H), 6.28 (d, *J* = 2.1 Hz, 1H), 4.24 (dd, *J* = 12.0, 4.2 Hz, 1H), 4.13 (dd, *J* = 12.0, 4.4 Hz, 1H), 3.79 (s, 3H), 2.79 – 2.70 (m, 1H), 1.51 (s, 3H), 1.41 (s, 3H).

**^13^C NMR (101 MHz, CDCl_3_)** δ 191.6, 169.5, 165.9, 163.2, 151.9, 131.3, 128.9, 127.5, 126.3, 116.0, 110.0, 100.4, 69.5, 55.6, 54.7, 40.4, 28.8, 24.1.

**HRMS** (ESI+): calculated m/z [M+H]^+^ for [C_20_H_22_NO_4_]^+^: 340.1543, found: 340.1545.

- 1. **The synthesis of 4-aryl-3-benzylchromene** **(7)**

To a solution of 4-(2-(7-methoxy-4-oxochroman-3-yl)propan-2-yl)benzonitrile) (**3cm**) (0.25 mmol, 1 equiv.) in dichloromethane (2 mL) were added 2,6-di-*tert*-butyllpyridine (0.5 mmol, 2 equiv.) and trifluoromethane sulfonic anhydride (0.5 mmol, 2 equiv.) at 0 °C, followed by stirring at the same temperature for 10 minutes, and then stirring at room temperature for additional 5 hours. The reaction mixture was diluted with hexane, and the insoluble materials were then separated by filtration, and the filtrate was concentrated under reduced pressure. The resulting residue was purified by silica gel column chromatography to afford the desired product **3cm'** in 93% yield.

A mixed solution of **3cm'** (0.23 mmol, 1 equiv.), *p*-tolylboronic acid (0.46 mmol, 2 equiv.), tetrakis(triphenylphosphine)palladium (10.6 mg, 4 mmol %), and DIPEA (0.46 mmol, 2 equiv.) in NMP (5 mL) was heated under stirring at 170 °C for 10 minutes. The reaction mixture was returned to room temperature, diluted with water, and then extracted with ethyl acetate. The organic layer was washed with water, dried, and then concentrated under reduced pressure. The resulting residue was purified by silica gel column chromatography to afford the desired product **7** in 86% yield.

**4-(2-(7-methoxy-4-(*p*-tolyl)-2*H*-chromen-3-yl)propan-2-yl)benzonitrile (7)**

**Physical State:** yellow oil.

**Yield:** 80%.

**^1^H NMR (400 MHz, CDCl_3_)** δ 7.57 – 7.50 (m, 2H), 7.40 (d, *J* = 8.5 Hz, 2H), 7.11 (d, *J* = 7.7 Hz, 2H), 6.99 (d, *J* = 8.0 Hz, 2H), 6.44 – 6.38 (m, 2H), 6.31 (dd, *J* = 8.6, 2.6 Hz, 1H), 4.41 (s, 2H), 3.74 (s, 3H), 2.38 (s, 3H), 1.27 (s, 6H).

**^13^C NMR (101 MHz, CDCl_3_)** δ 160.2, 154.9, 154.6, 136.7, 135.2, 132.5, 132.2, 131.8, 130.0, 128.7, 127.6, 127.0, 120.1, 119.0, 109.8, 107.3, 100.8, 67.8, 55.4, 45.1, 29.7, 21.3.

**HRMS** (ESI+): calculated m/z [M+H]^+^ for [C_27_H_26_NO_2_]^+^: 396.1958, found: 396.1967.

- 1. **The synthesis of β-pyridylated ketone (8)**

An oven-dried Schlenk tube (10 mL) equipped with a stirring bar was charged with P(*p*-tol)_3_ (0.8 mmol, 4 equiv.), 3DPAFIPN (0.004 mmol, 2 mol%), 4-cyanopyridine (0.2 mmol, 1 equiv.), benzoic acid (0.4 mmol, 2 equiv.), K_2_HPO_4_ (0.6 mmol, 3 equiv.). The tube was connected to a vacuum line where it was evacuated and back-filled with Ar for three times. Then, styrene (0.3 mmol, 1.5 equiv.) and MeCN (4.0 mL) were added under Ar flow. The tube was placed approximately 2 cm from 30 W blue LEDs, and stirred at room temperature for 48 h. After completion, the mixture was concentrated in vacuo. The resulting residue was purified by flash column chromatography to afford the desired product **8** in 35% yield.

**1,3-diphenyl-3-(pyridin-4-yl)propan-1-one (8)**

**Physical State:** yellow oil.

**Yield:** 35%.

**^1^H NMR (400 MHz, CDCl_3_)** δ 8.49 (d, *J* = 5.2 Hz, 2H), 7.95 (d, *J* = 7.4 Hz, 2H), 7.58 (t, *J* = 7.4 Hz, 1H), 7.46 (t, *J* = 7.7 Hz, 2H), 7.35 – 7.28 (m, 2H), 7.28 – 7.22 (m, 3H), 7.19 (d, *J* = 5.9 Hz, 2H), 4.86 – 4.77 (m, 1H), 3.84 – 3.66 (m, 2H).

**^13^C NMR (101 MHz, CDCl_3_)** δ 197.2, 153.0, 149.9, 142.5, 136.7, 133.4, 128.9, 128.7, 128.0, 127.9, 127.0, 123.2, 45.2, 43.9.

**HRMS** (ESI+): calculated m/z [M+H]^+^ for [C_20_H_18_NO]^+^: 288.1383, found: 288.1392.

1. **Mechanistic studies**
   1. **Radical-inhibition experiments**

An oven-dried Schlenk tube (10 mL) equipped with a stirring bar was charged with P(*p*-tol)_3_ (0.2 mmol, 2.0 equiv.), 3DPAFIPN (0.002 mmol, 2 mol%), the corresponding cyanoarene **2a** (0.15 mmol, 1.5 equiv), TEMPO (0.3 mmol, 3 equiv.). The tube was connected to a vacuum line where it was evacuated and back-filled with Ar for three times. Then, alkenoic acid **1a** (0.1 mmol, 1.0 equiv.) and MeCN (2.0 mL) were added under Ar flow. The tube was placed approximately 2 cm from 30 W blue LEDs and stirred at room temperature for 24 h. After completion, the corresponding **1a**-derived TEMPO-trapped adduct **TEMPO-1a** was detected through ESI-HRMS analysis, and the desired product **3aa** was not detected. **HRMS (ESI)** calcd for **TEMPO-1a** C_21_H_32_NO_3_[M+H]^+^: 346.2377, found: 346.2390.

**Figure S1.** The ESI-HRMS spectrum of the corresponding **TEMPO**-trapped adduct **TEMPO**-**1a**.

- 1. **Electron-transfer scavenging experiment**

An oven-dried Schlenk tube (10 mL) equipped with a stirring bar was charged with P(*p*-tol)_3_ (0.2 mmol, 2.0 equiv.), 3DPAFIPN (0.002 mmol, 2 mol%), the corresponding cyanoarene **2a** (0.15 mmol, 1.5 equiv), DNB (0.3 mmol, 3 equiv.). The tube was connected to a vacuum line where it was evacuated and back-filled with Ar for three times. Then, alkenoic acid **1a** (0.1 mmol, 1.0 equiv.) and MeCN (2.0 mL) were added under Ar flow. The tube was placed approximately 2 cm from 30 W blue LEDs and stirred at room temperature for 24 h. After completion, the desired product **3aa** was not detected.

- 1. **Competitive experiment**

An oven-dried Schlenk tube (10 mL) equipped with a stirring bar was charged with P(*p*-tol)_3_ (0.2 mmol, 2.0 equiv.), 3DPAFIPN (0.002 mmol, 2 mol%), the corresponding cyanoarene **2a** (0.15 mmol, 1.5 equiv), DPE (0.3 mmol, 3 equiv.). The tube was connected to a vacuum line where it was evacuated and back-filled with Ar for three times. Then, alkenoic acid **1a** (0.1 mmol, 1.0 equiv.) and MeCN (2.0 mL) were added under Ar flow. The tube was placed approximately 2 cm from 30 W blue LEDs and stirred at room temperature for 24 h. After completion, only a trace amount of desired product **3aa** was detected by ^1^H-NMR spectroscopy using 1,3,5-trimethoxybenzene (16.8 mg, 0.1 mmol) as internal standard, and the addition products **3aa'** was captured by ESI-HRMS analysis. **HRMS (ESI)** calcd for **3aa'** C_26_H_25_O_2_ [M+H]^+^: 369.1849, found: 369.1853.

**Figure S2.** The ESI-HRMS spectrum of the corresponding DPE addition products **3aa'**.

- 1. **Cyclic voltammetry test**

Cyclic voltammetry test was performed in a three-electrode cell under argon at room temperature. All cyclic voltammograms were measured using Ag/Ag^+^ (0.01 M AgNO_3_ in MeCN) reference electrode, platinum (Pt) wire counter electrode, and a glassy carbon working electrode. The conditions of the experiments were as follows: testing compounds are in a solution of 0.1 M hexafluorophosphate (*n-*Bu_4_NPF_6_) in CH_3_CN at a scan rate of 50 mV/s; Prior to each measurement, solutions were purged with argon for 10 minutes to ensure the oxygen-free conditions.

Measuring the Fc/Fc^+^ redox couple afforded *E*_1/2_ = +0.07 V vs Ag/Ag^+^ under our experimental conditions. The obtained value was referenced to Ag/Ag^+^ and converted to SCE by subtracting 0.35 V, providing a value of +0.42 V for the Fc/Fc^+^ couple.(Roth et al., 2016) The oxidation half-peak potential of P(*p*-tol)_3_ in MeCN was measured as +0.68 V (*vs.* Ag/Ag^+^), and calculated to +1.03 V (*vs.* SCE).


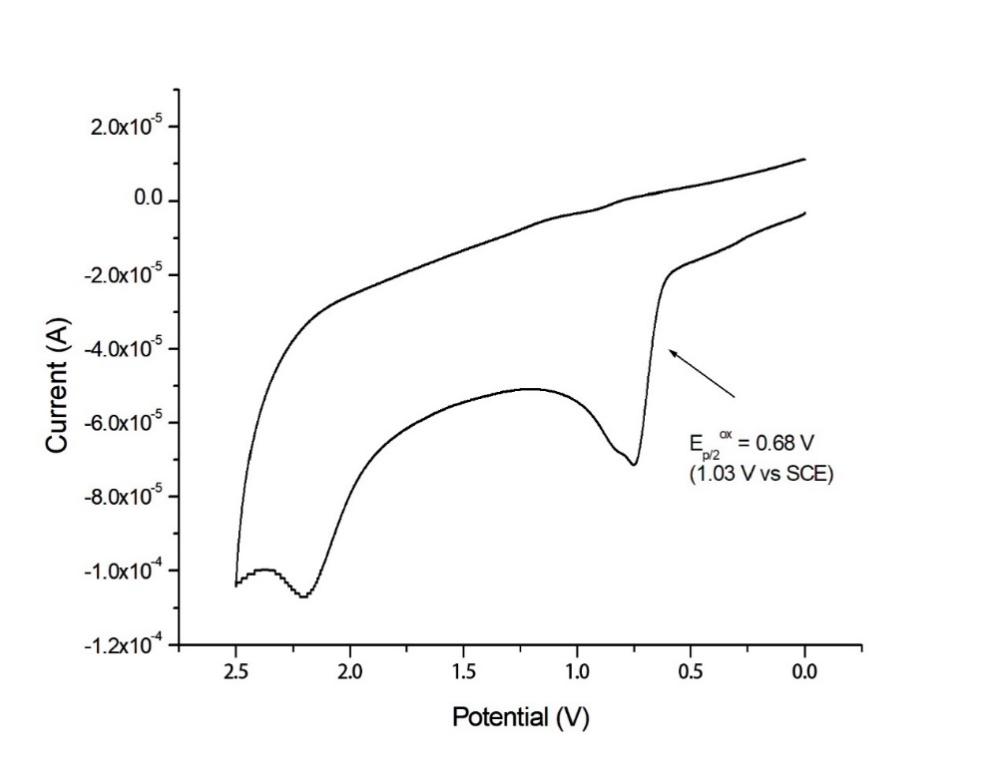


**Figure S3.** Cyclic voltammetry of P(*p*-tol)_3_ (5 mM) in MeCN (*vs*. Ag/Ag^+^) with ^n^Bu_4_NPF_6_ (0.1 M) under argon at a glassy carbon electrode at a scan rate of 50 mV/s.


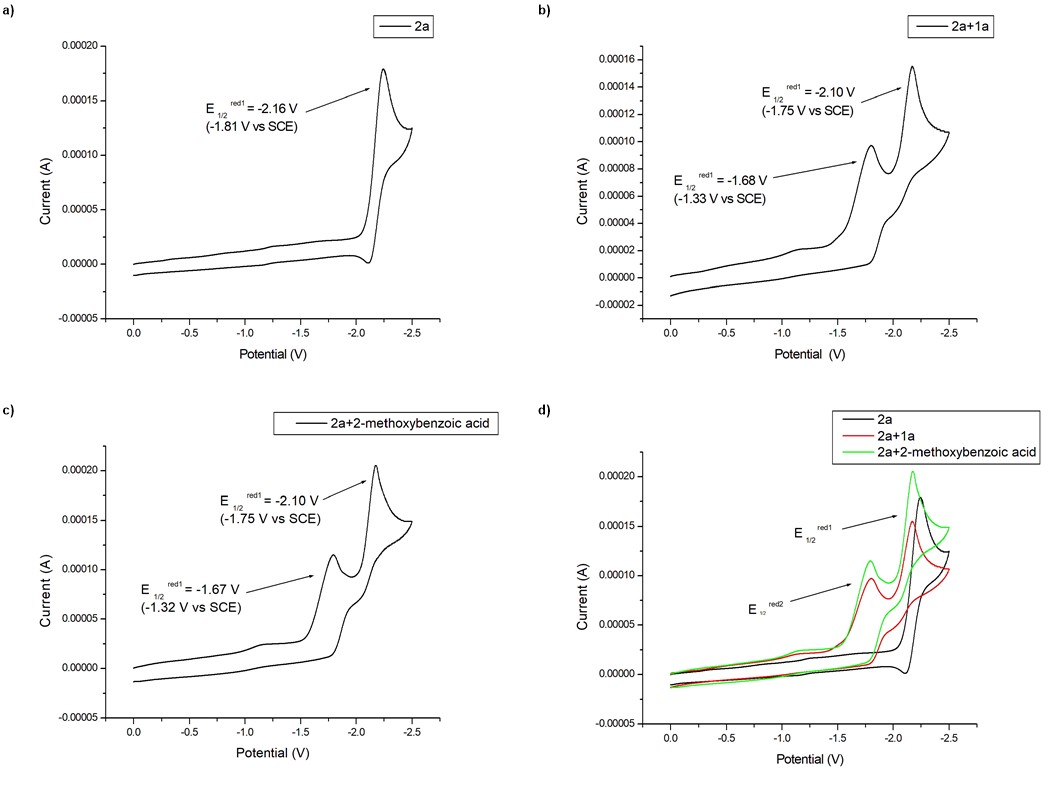
**Figure S4.** a) Cyclic voltammetry of **2a** (5 mM) in MeCN (*vs*. Ag/Ag^+^) with ^n^Bu_4_NPF_6_ (0.1 M) under argon at a glassy carbon electrode at a scan rate of 50 mV/s; b) Cyclic voltammetry of **2a** (5 mM) and **1a** (5 mM) in MeCN (*vs*. Ag/Ag^+^) with ^n^Bu_4_NPF_6_ (0.1 M) under argon at a glassy carbon electrode at a scan rate of 50 mV/s; c) Cyclic voltammetry of **2a** (5 mM) and 2-methoxybenzoic acid (5 mM) in MeCN (*vs*. Ag/Ag^+^) with ^n^Bu_4_NPF_6_ (0.1 M) under argon at a glassy carbon electrode at a scan rate of 50 mV/s; d) Cyclic voltammetry of **2a** (5 mM) with different carboxylic acids.

- 1. **^1^H NMR experiments**


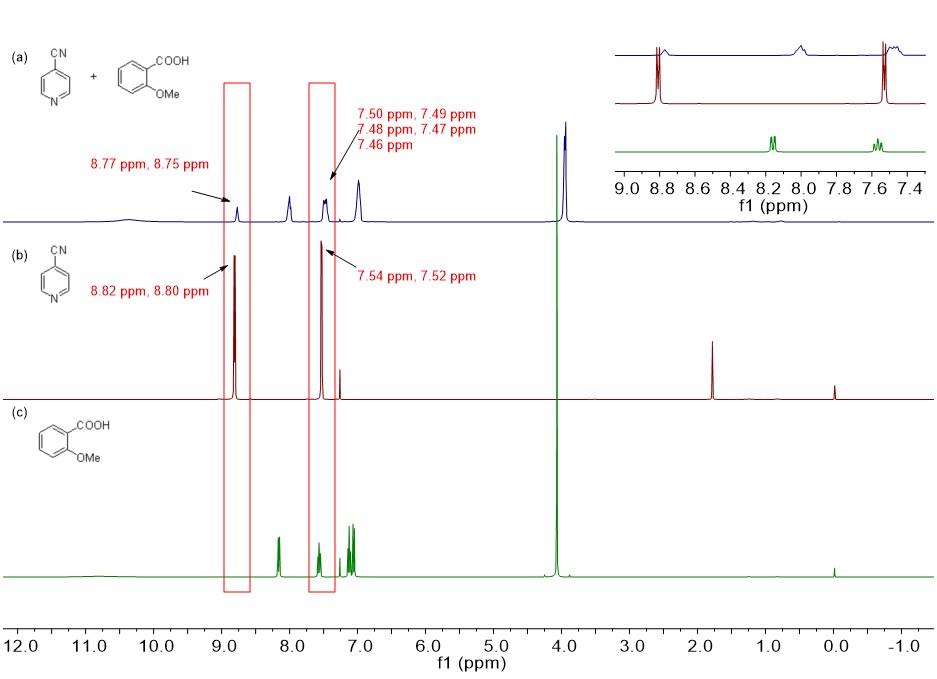


**Figure S5.** ^1^H NMR experiments (400 MHz, CDCl_3_ with TMS, recorded at 25 °C): (a) **2a** with 2-methoxybenzoic acid (5.0 equiv); (b) **2a** (10 mg); (c) 2-methoxybenzoic acid (15 mg).

1. **X-ray crystal data**
   1. **X-ray crystal data of 3ga**

**
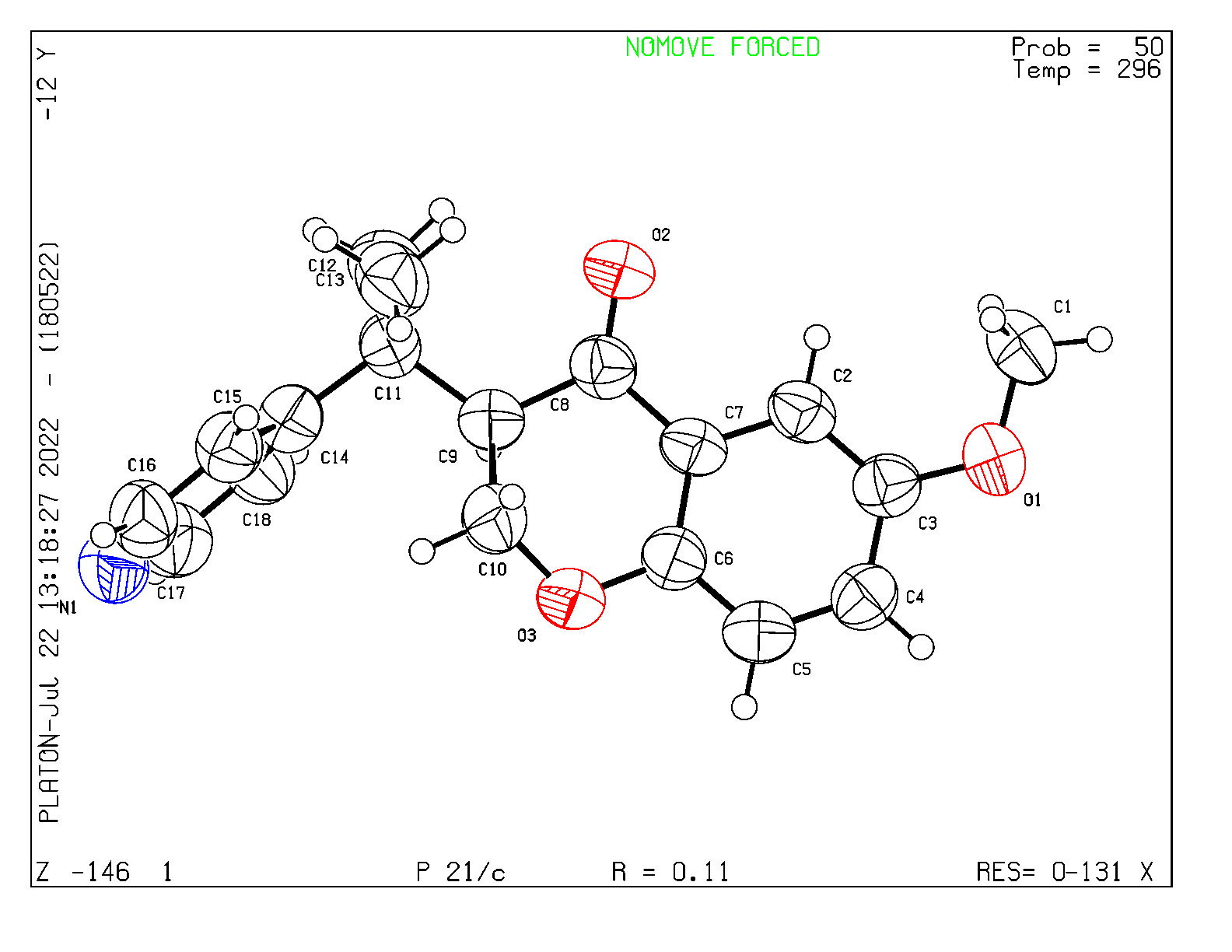
**

**Figure S6.** X-ray crystal structure of **3ga**

(The crystal was obtained by slow evaporation of the solution of CH_2_Cl_2_ and hexane) (CCDC 2192065):

| Bond precision: | C-C = 0.0084 A | | Wavelength=0.71073 |
| --- | --- | --- | --- |
| Cell: | a=13.20(3) | b=10.61(2) | c=10.83(3) |
|  | alpha=90 | beta=94.76(5) | gamma=90 |
| Temperature: | 296 K |  |  |
|  | **Calculated** | | **Reported** |
| Volume | 1512(6) | | 1511(6) |
| Space group | P 21/c | | P 21/c |
| Hall group | -P 2ybc | | -P 2ybc |
| Moiety formula | C18 H19 N O3 | | ? |
| Sum formula | C18 H19 N O3 | | C18 H19 N O3 |
| Mr | 297.34 | | 297.34 |
| Dx,g cm-3 | 1.306 | | 1.307 |
| Z | 4 | | 4 |
| Mu (mm-1) | 0.089 | | 0.089 |
| F000 | 632.0 | | 632.0 |
| F000' | 632.30 | |  |
| h,k,lmax | 15,12,12 | | 15,12,12 |
| Nref | 2661 | | 2618 |
| Tmin,Tmax | 0.982,0.982 | | 0.864,0.864 |
| Tmin' | 0.982 | |  |
| Correction method= # Reported T Limits: Tmin=0.864 Tmax=0.864 AbsCorr = MULTI-SCAN | | | |
| Data completeness= 0.984 | | | Theta(max)= 24.998 |
| R(reflections)= 0.1054( 1115) | | | wR2(reflections)= 0.2495( 2618) |
| | S = 1.150 | Npar= 202 | | --- | --- | | | |  |

- 1. **X-ray crystal data of 3ah**

**
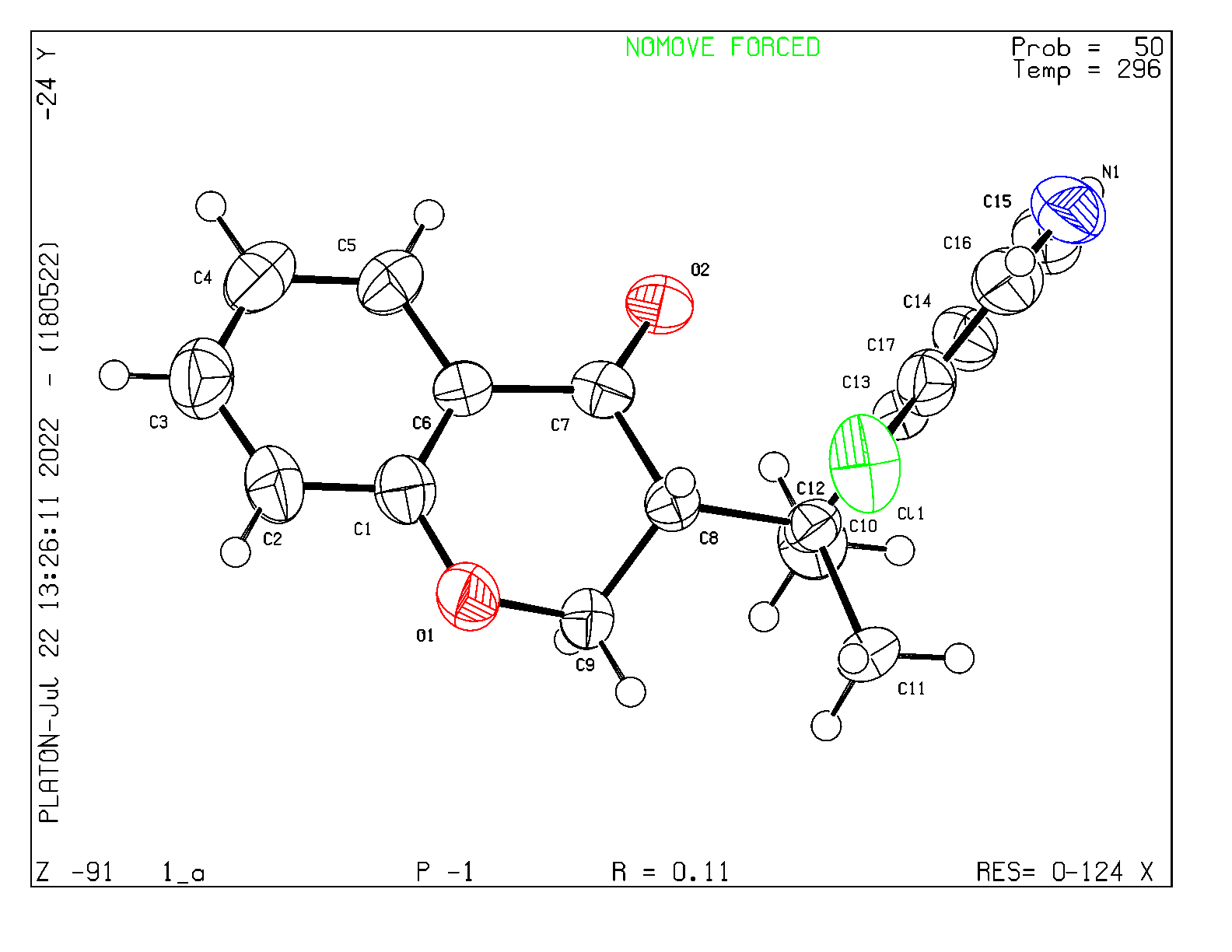
**

**Figure S7.** X-ray crystal structure of **3ah**

(The crystal was obtained by slow evaporation of the solution of CH_2_Cl_2_ and hexane) (CCDC 2192094):

| Bond precision: | C-C = 0.0088 A | | Wavelength=0.71073 | |
| --- | --- | --- | --- | --- |
| Cell: | a=6.95700 | b=7.62100 | c=14.24400 | |
|  | alpha=96.4200 | beta=90.0200 | gamma=106.7500 | |
| Temperature: | 296 K |  |  | |
|  | **Calculated** | | **Reported** | |
| Volume | 718.208 | | 718 | |
| Space group | P -1 | | P -1 | |
| Hall group | -P 1 | | -P 1 | |
| Moiety formula | C17 H16 Cl N O2 | | ? | |
| Sum formula | C17 H16 Cl N O2 | | C17 H16 Cl N O2 | |
| Mr | 301.76 | | 301.76 | |
| Dx,g cm-3 | 1.395 | | 1.395 | |
| Z | 2 | | 2 | |
| Mu (mm-1) | 0.270 | | 0.270 | |
| F000 | 316.0 | | 316.0 | |
| F000' | 316.42 | |  | |
| h,k,lmax | 8,9,16 | | 0,0,0 | |
| Nref | 2525 | | 2494 | |
| Tmin,Tmax | 0.947,0.947 | | 0.864,0.864 | |
| Tmin' | 0.947 | |  | |
| Correction method= # Reported T Limits: Tmin=0.864 Tmax=0.864 AbsCorr = MULTI-SCAN | | | |
| Data completeness= 0.988 | | | Theta(max)= 25.000 |
| R(reflections)= 0.1123( 1543) | | | wR2(reflections)= 0.2909( 2494) |
| | S = 1.058 | Npar= 180 | | --- | --- | | | |

1. **Supplementary references**

Carrillo-Arcos, U.A., Rojas-Ocampo, J., and Porcel, S. (2016). Oxidative cyclization of alkenoic acids promoted by agoac. *Dalton Trans.* 45, 479–483. doi:10.1039/C5DT03808A

Chen, H., Jin, W., and Yu, S. (2020). Enantioselective remote C(sp3)-H cyanation via dual photoredox and copper catalysis. *Org. Lett.* 22, 5910–5914. doi:10.1021/acs.orglett.0c02008

De, S., and Rigby, J.H. (2013). Total synthesis of (±)-debromoflustramine B via [4+1] cyclization of a bis(alkylthio)carbene and an indole isocyanate. *Tetrahedron Lett.* 54, 4760–4762. doi:10.1016/j.tetlet.2013.06.119

Hoang, G.L., Zhang, S., and Takacs, J.M. (2018). Rhodium-catalyzed asymmetric hydroboration of γ,δ-unsaturated amide derivatives: Δ-borylated amides. *Chem. Commun.* 54, 4838-4841. doi:10.1039/C8CC01563E

Li, J., Lu, X.-C., Xu, Y., Wen, J.-X., Hou, G.-Q., and Liu, L. (2020). Photoredox catalysis enables decarboxylative cyclization with hypervalent iodine(III) reagents: Access to 2,5-disubstituted 1,3,4-oxadiazoles. *Org. Lett.* 22, 9621–9626. doi:10.1021/acs.orglett.0c03663

Roth, H.G., Romero, N.A., and Nicewicz, D.A. (2016). Experimental and calculated electrochemical potentials of common organic molecules for applications to single-electron redox chemistry. *Synlett* 27, 714–723.

Speckmeier, E., Fischer, T.G., and Zeitler, K. (2018). A toolbox approach to construct broadly applicable metal-free catalysts for photoredox chemistry: Deliberate tuning of redox potentials and importance of halogens in donor-acceptor cyanoarenes. *J. Am. Chem. Soc.* 140, 15353–15365. doi:10.1021/jacs.8b08933

1. **Copies of NMR spectra for products**

^1^H NMR spectrum of **3aa**

^13^C NMR spectrum of **3aa**

^1^H NMR spectrum of **3ba**

^13^C NMR spectrum of **3ba**

^1^H NMR spectrum of **3ca**

^13^C NMR spectrum of **3ca**

^1^H NMR spectrum of **3da**

^13^C NMR spectrum of **3da**

^1^H NMR spectrum of **3ea**

^13^C NMR spectrum of **3ea**

^19^F NMR spectrum of **3ea**

^1^H NMR spectrum of **3fa**

^13^C NMR spectrum of **3fa**

^19^F NMR spectrum of **3fa**

^1^H NMR spectrum of **3ga**

^13^C NMR spectrum of **3ga**

^1^H NMR spectrum of **3ha**

^13^C NMR spectrum of **3ha**

^1^H NMR spectrum of **3ia**

^13^C NMR spectrum of **3ia**

^1^H NMR spectrum of **3ja**

^13^C NMR spectrum of **3ja**

^19^F NMR spectrum of **3ja**

^1^H NMR spectrum of **3ka** (major diastereomer)

^13^C NMR spectrum of **3ka** (major diastereomer)

^1^H NMR spectrum of **3ka** (minor diastereomer)

^13^C NMR spectrum of **3ka** (minor diastereomer)

^1^H NMR spectrum of **3la**

^13^C NMR spectrum of **3la**

^1^H NMR spectrum of **3ma**

^13^C NMR spectrum of **3ma**

^1^H NMR spectrum of **3na**

^13^C NMR spectrum of **3na**

^1^H NMR spectrum of **3oa**

^13^C NMR spectrum of **3oa**

^1^H NMR spectrum of **3pa**

^13^C NMR spectrum of **3pa**

^1^H NMR spectrum of **3qa**

^13^C NMR spectrum of **3qa**

^1^H NMR spectrum of **3ra**^13^C NMR spectrum of **3ra**^1^H NMR spectrum of **3sa**

^13^C NMR spectrum of **3sa**

^1^H NMR spectrum of **3ta** ^13^C NMR spectrum of **3ta**

^1^H NMR spectrum of **3ab**

^13^C NMR spectrum of **3ab**

^1^H NMR spectrum of **3ac**

^13^C NMR spectrum of **3ac**

^1^H NMR spectrum of **3ad**

^13^C NMR spectrum of **3ad**

^1^H NMR spectrum of **3ae**

^13^C NMR spectrum of **3ae**

^19^F NMR spectrum of **3ae**

^1^H NMR spectrum of **3af**

^13^C NMR spectrum of **3af**

^1^H NMR spectrum of **3ag**

^13^C NMR spectrum of **3ag**

^19^F NMR spectrum of **3ag**

^1^H NMR spectrum of **3ah**

^13^C NMR spectrum of **3ah**

^1^H NMR spectrum of **3ai**

^13^C NMR spectrum of **3ai**

^19^F NMR spectrum of **3ai**

^1^H NMR spectrum of **3aj**

^13^C NMR spectrum of **3aj**

^1^H NMR spectrum of **3aj'**

^13^C NMR spectrum of **3aj'**

^1^H NMR spectrum of **3ak**

^13^C NMR spectrum of **3ak**

^1^H NMR spectrum of **3al**

^13^C NMR spectrum of **3al**

^1^H NMR spectrum of **3cm**

^13^C NMR spectrum of **3cm**

^1^H NMR spectrum of **4**

^13^C NMR spectrum of **4**

^1^H NMR spectrum of **5**

^13^C NMR spectrum of **5**

^1^H NMR spectrum of **6**

^13^C NMR spectrum of **6**

^1^H NMR spectrum of **7** ^13^C NMR spectrum of **7**

^1^H NMR spectrum of **8**

^13^C NMR spectrum of **8**
